# Socio-economic factors constrain climate change adaptation in a tropical export crop

**DOI:** 10.1038/s43016-025-01130-1

**Published:** 2025-03-06

**Authors:** Varun Varma, Jonathan R. Mosedale, José Antonio Guzmán Alvarez, Daniel P. Bebber

**Affiliations:** 1 Rothamsted Research, Harpenden, UK; 2 Department of Biosciences, University of Exeter, Exeter, UK; 3 Environmental & Sustainability Institute, University of Exeter, Penryn, UK; 4 CORBANA, San José, Zapote Costa Rica; 5 Global Systems Institute, University of Exeter, Exeter, UK

**Keywords:** Climate-change adaptation, Agroecology, Climate-change ecology

## Abstract

Climate change will alter the geographical locations most suited for crop production, but adaptation to these new conditions may be constrained by edaphic and socio-economic factors. Here we investigate climate change adaptation constraints in banana, a major export crop of Latin America and the Caribbean. We derived optimal climatic, edaphic and socio-economic conditions from the distribution of intensive banana production across Latin America and the Caribbean, identified using remote sensing imagery. We found that intensive banana production is constrained to low-lying, warm aseasonal regions with slightly acidic soils, but is less constrained by precipitation, as irrigation facilitates production in drier regions. Production is limited to areas close to shipping ports and with high human population density. Rising temperatures, coupled with requirements for labour and export infrastructure, will result in a 60% reduction in the area suitable for export banana production, along with yield declines in most current banana producing areas.

## Abstract (web summary)

Understanding and preparing for the impacts of climate change on tropical crops is key for global food security and sustainable development, especially in the Global South. This study maps banana production in Latin America and the Caribbean in 2019, and analyses the potential impact of climate change and other factors on banana producing regions.

## Main

Climate is a major determinant of global vegetation distributions^1^. As climate changes, the geographical regions suitable for different vegetation types will follow^2^. The influence of climate extends to the geographical distributions of agricultural crops^3^ and how these may change in future^4^. There are several key differences between the climate change responses of natural plant populations and those of agricultural crops. Humans alter both the climatic niche of crop plants through breeding^5^ and the suitability of habitat through technologies such as irrigation^6^. Socio-economic factors such as local market preferences, export market access and government subsidies also influence farmer choice in selecting crops for production^7^. Adaptation of agriculture to climate change must therefore also consider social, economic and cultural constraints to crop production.

The capacity for climate change adaptation may be particularly limited in the tropics, for several reasons. First, unlike higher latitudes, which may become suitable for warm-adapted crops over time^8^, equatorial regions already experience the warmest temperatures and thus lack regions from which novel crops could be sourced. Plant breeding for heat tolerance^9^, increased reliance on the most heat-tolerant crops and increased deployment of technologies such as irrigation could mitigate this limitation to some degree. Second, warming in the tropics may not enhance yields^10^ or extend the growing season of crops already present^11^, as expected in extratropical regions. Third, the global latitudinal trend in wealth and technological capacity^12^ means that farmers in the Global South may be less able to adapt agricultural practices to cope with changing climate than their counterparts in wealthier countries^13^. Understanding and preparing for the impacts of climate change on tropical crops is therefore of central concern in ensuring global food security and sustainable development, particularly where populations are expected to grow rapidly^14^.

Here we analyse the potential impact of climate change on the production of a major tropical export crop, banana. Bananas and closely related plantains are the fruit of various hybrids and cultivars of *Musa* species^15^. While banana species were domesticated in Southeast Asia to produce a huge diversity of edible varieties, the international banana trade relies on a single cultivar, Cavendish^16^. Around 50 million tonnes of Cavendish bananas (comprising a number of somaclonal cultivars) are produced each year, approximately half of global banana production. Of this, 20 million tonnes are internationally traded. Europe and the United States are the biggest importers, sourcing the majority of their bananas from Latin America. This global trade is worth around US$11 billion, exceeding that of any other exported fruit^17^. Banana exports are therefore an important source of revenue for certain low- and middle-income countries. In Colombia, for example, the industry accounts for around 5% of agricultural gross domestic product and employs nearly 300,000 workers directly or indirectly^18^. As a tropical species, bananas are able to grow under hot conditions but are highly sensitive to water deficits^19^. Bananas are therefore potentially vulnerable to climate change where temperatures exceed optima and where water is limiting, but they may benefit where warming approaches growth optima and water is sufficient^20,21^. Unfortunately, detailed ecophysiological information on banana productivity in relation to climate is scarce^19,22^, and the available geographical distribution data are based on probabilistic estimates rather than observation^23^, hampering efforts to understand how climate change might affect this crop.

To facilitate our analysis, we developed a high-resolution map of banana production for Latin America and the Caribbean for the year 2019. We refer to this dataset as BM19 (Banana Map 2019) hereafter. Previous studies of banana production have employed subnational production data^20^ or visual identification of banana plantations in Google Earth images^21^ to infer climatic tolerances for this crop. The latter approach is possible because of certain characteristics of bananas in comparison with other crops and vegetation types: banana plants are tall with very large leaves and uniform canopies, and tend to be planted with quasi-regular spacing. Furthermore, bananas for export are grown in a continuous production cycle that leaves a characteristic temporal signal in satellite data. These spatio-temporal attributes have enabled detailed mapping of hurricane impacts on banana plantation production in the Dominican Republic using a combination of synthetic aperture radar (SAR) and multi-spectral remote sensing^24^. An important caveat is that a large fraction of global banana production is by smallholders, often in sparser, mixed cropping systems in which the banana canopy is difficult to distinguish from surrounding vegetation except at very high spatial resolution^25^. Hence, we restrict our analysis to high-density export banana production, as in previous research^21^. Because of the reliance of export banana production on manual labour, access to shipping ports and irrigation in drier areas^16^, we quantify the degree to which banana plantation distributions are restricted to areas with high population density, near ports and equipped for irrigation. We project future suitability for banana production under climate change and assess the influence of socio-economic factors in constraining potential responses to climate change through relocation of production.

## Results

### Banana map BM19

Our classifier proved adept at identifying banana plantations (Supplementary Table 2). All 500 plantation grid cells in the test dataset were correctly identified, and only 20 of the remaining 2,500 grid cells of other land-use classes were misclassified as banana, of which 17 were mangrove. Young and developing plantations with sparse canopies were not identified as banana plantations.

**Fig. 1:**
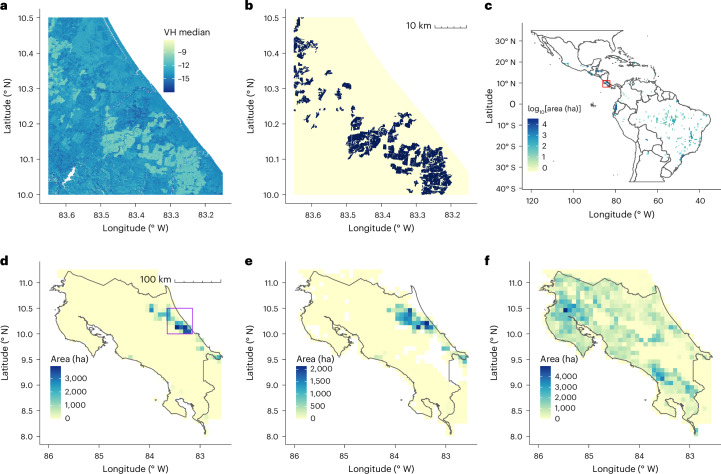
Banana distribution maps. **a**, Sentinel-1 median SAR VH (vertical transmit, horizontal receive polarization) for 2019 over the Caribbean coast of Costa Rica, aggregated to 100-m resolution. **b**, BM19 banana presence (black) over the same region, 50-m resolution. **c**, BM19 for Latin America and the Caribbean, aggregated to ha per 0.5° grid cell. The colour scale is log-transformed. The axes are in degrees. The red square indicates Costa Rica. **d**, BM19 banana area in Costa Rica aggregated to ha per five-arcminute grid cell. The purple square outlines the Caribbean coast region in **a** and **b**. **e**, SPAM banana area in Costa Rica. **f**, GAEZ banana area in Costa Rica. Source data

**Extended Data Fig. 1:**
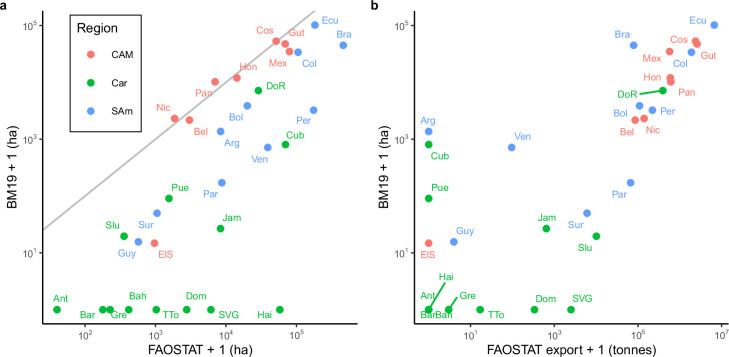
BM19 compared with FAOSTAT. **a**. BM19 vs. FAOSTAT banana area by country and region (Car = Caribbean, CAM = Central America and Mexico, SAm = South America) for 2019. Log-transformed axes were used therefore 1 ha was added to enable zero values to be visualized. Country codes are given in Table S1. **b**. BM19 banana area vs. FAOSTAT banana export by country and region for 2019. Source data

**Extended Data Fig. 2:**
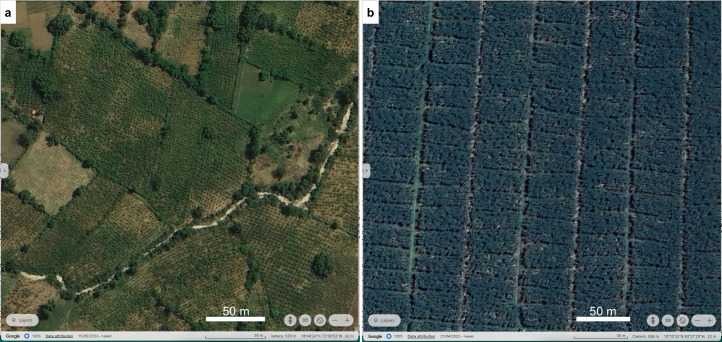
Aerial imagery of banana production. **a**. Putative banana or plantain production in the Archahaie Arrondissement to the north west of Porte-au-Prince. Aerial imagery obtained from Google Earth (https://earth.google.com). Data from Airbus (2023). **b**. High intensity export banana production near San Cristobal, Costa Rica. Aerial imagery obtained from Google Earth (https://earth.google.com). Data from CNES/Airbus (2022).

High levels of Sentinel-1 SAR backscatter were associated with banana (Fig. 1a,b). The classifier identified 360,662 ha of banana plantations >0.5 ha within the region of interest (ROI) (Fig. 1c). The detected area of plantations equates to 24.4% of the total harvested banana area listed by FAOSTAT in countries within the ROI for 2019 (Supplementary Table 3). The total BM19 banana area is closest to FAOSTAT estimates for Central American countries, Mexico, Ecuador, Colombia and the Dominican Republic (Extended Data Fig. 1a). However, the BM19 classifier detected no banana plantations >0.5 ha in several Caribbean states that report large banana production areas, including Haiti, St. Vincent and the Grenadines, Dominica, and Trinidad and Tobago (Supplementary Table 3). Banana area in Haiti is reported to be slightly larger than that in Costa Rica and is concentrated in the Archahaie Arrondissement to the northwest of Porte-au-Prince^26^. We were unable to obtain ground-truth verification of banana production in Haiti. Inspection of aerial imagery and expert opinion (T. Lescot, personal communication) suggests a far sparser production system in Haiti than in commercial export plantations (Extended Data Fig. 2), though this interpretation is uncertain (R. Yudin, personal communication).

**Extended Data Fig. 3:**
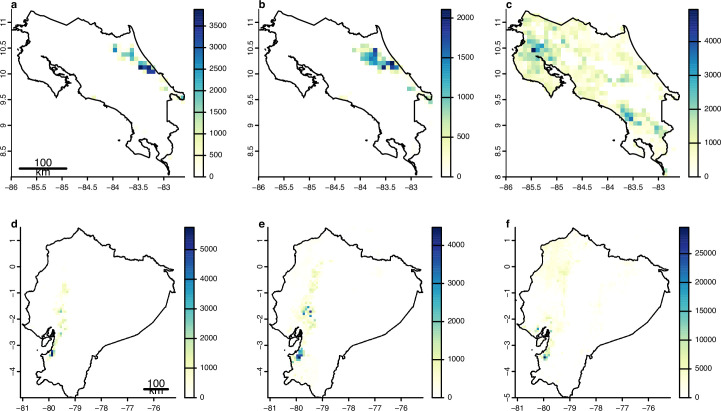
Banana area estimate comparison. Upper row (**a**-**c**) is Costa Rica, lower row (**d**-**f**) is Ecuador. Left column (**a**,**d**) is BM19, centre (**b**,**e**) is SPAM, right (**c**,**f**) is GAEZ. Spatial resolution 5 arc minutes.

BM19 area is strongly correlated with banana export quantity for Central American countries, Mexico, Belize, Colombia, the Dominican Republic, Bolivia and Peru (Extended Data Fig. 1b). Some Caribbean islands including St. Vincent and the Grenadines have high export quantities though no banana production was detected by the BM19 classifier, while Argentina, Cuba, Puerto Rico and El Salvador report no exports but have substantial banana production. BM19 banana area tends to have a more spatially restricted distribution than both the Spatial Production Allocation Model (SPAM) 2010 and the Global Agro-Ecological Zones (GAEZ+ 2015) harvested area estimates derived from the spatial downscaling of national and subnational production statistics (Fig. 1d–f and Extended Data Fig. 3). Of the two derived products, BM19 more closely resembles the SPAM distribution with more localized and intensely cultivated areas than GAEZ.

### Climatic, edaphic and socio-economic limits of banana plantations

**Fig. 2:**
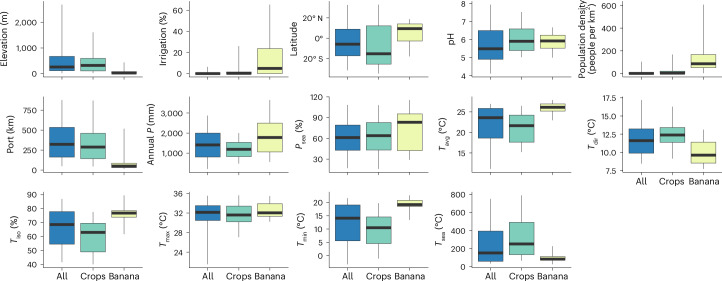
Banana plantation distributions in relation to climatic, edaphic and socio-economic variables. The boxes show the area interquartile range (with the median), and the whiskers show *R*_90_ of banana-area-weighted grid cells for the entire ROI (all), croplands (crops) and BM19 (banana) for elevation, area equipped for irrigation, latitude, soil pH at 0–5 cm, population density, distance to nearest port (Port), annual precipitation (*P*), precipitation seasonality (*P*_sea_), mean annual temperature (*T*_avg_), temperature diurnal range (*T*_dir_), temperature isothermality (*T*_iso_), maximum temperature of the warmest month (*T*_max_), minimum temperature of the coldest month (*T*_min_) and temperature seasonality (*T*_sea_). The sample size (the number of land area grid cells) is 261,871. Source data

Banana plantations tended to be restricted to particular climatic, edaphic and socio-economic conditions (Fig. 2 and Supplementary Table 3). Banana plantations were found at lower elevations (95th percentile of area below 440 m above sea level) and in more acidic soils (90th percentile of area between pH 5.0 and 6.7) than croplands in general. The latitudinal range was narrower than croplands, with somewhat greater annual precipitation, much narrower and higher annual mean and minimum temperatures, and much narrower and lower temperature seasonality. Precipitation seasonality and maximum temperature distributions were similar to but somewhat higher than those for other croplands. Banana was far more likely to be grown in areas equipped for irrigation than other crops, in areas with higher population density and much closer to ports: three quarters of mapped banana area lies within 86 km of the nearest port.

**Fig. 3:**
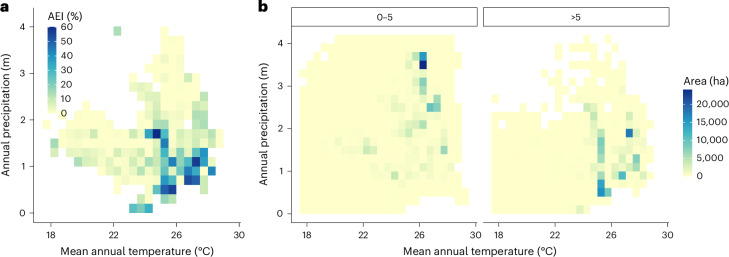
Area equipped for irrigation by mean annual temperature and annual precipitation. **a**, The colour scale shows the banana-area-weighted mean area equipped for irrigation (AEI) by climate. **b**, The colour scale shows the total banana area by climate, in regions with low (0–5% of area) irrigation (left) and high (>5% of area) irrigation (right). Source data

The requirement for irrigation tends to be greater in hotter, drier regions (Fig. 3a). Irrigation provision increases below 1,500 mm annual precipitation and above 25 °C mean annual temperature. An exception to this pattern is seen at around 25 °C and 1,700 mm precipitation, which has a very high (60%) area equipped for irrigation. This anomalously high irrigation provision is found near Guayaquil, Ecuador. Median irrigation provision, weighted by banana area per grid cell, is around 5% (Supplementary Table 4). Banana production area for low-irrigation areas (<5%) is concentrated at around 26 °C and 3,500 mm precipitation, while production in high-irrigation areas (≥5%) occurs above 25 °C and below 2,000 mm (Fig. 3b). The 90th percentile range (*R*_90_) of climatic variables for banana production differed by irrigation provision level (Supplementary Table 5). The median and *R*_90_ of mean annual temperatures (26.1, 22.9 and 27.9 °C) were similar to the optimum, minimum and maximum annual temperatures (26.8, 20.0 and 29.4 °C) estimated for Latin America and the Caribbean derived from a global banana yield analysis^20^. Total annual precipitation differed most strongly by irrigation, with nearly twice the rainfall found in low-irrigation versus high-irrigation areas (median 2,473 versus 1,210 mm).

### Climate change impacts on export banana production area

We used the observed distributions of banana plantations in relation to climatic, edaphic and socio-economic variables to estimate the potential geographical distributions under historical (1970–2000) and future (Shared Socio-economic Pathway (SSP) 2-4.5, 2061–2080) climates. For low-irrigation regions, we used the observed *R*_90_ for precipitation (912–3,690 mm). For high-irrigation regions, we lowered the minimum rainfall to 543 mm (the 5th percentile of the precipitation distribution for high-irrigation regions) but retained the upper limit at the low-irrigation value. The logic here is that banana production could occur under high precipitation in high-irrigation areas but that irrigation infrastructure tends not to be established where rainfall is plentiful.

**Extended Data Fig. 4:**
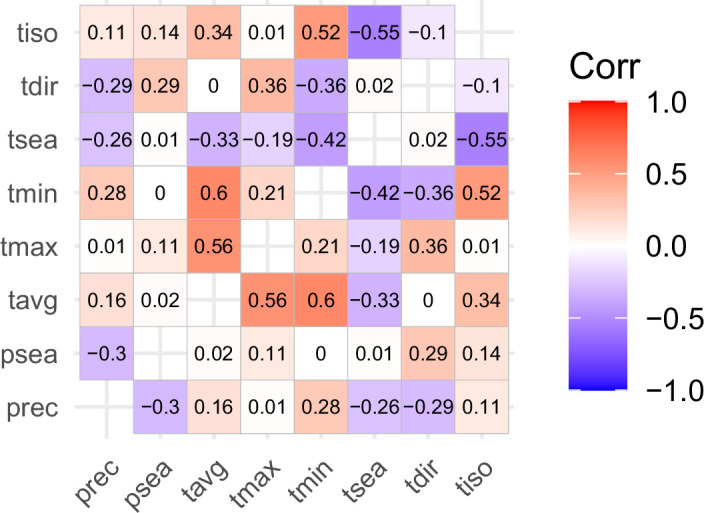
Kendall correlations among climate variables. Correlations for all 5 arc minute grid cells containing banana (n = 1203). Temperature variables are isothermality (tiso), mean diurnal range (tdir), seasonality (tsea), minimum (tmin), maximum (tmax) and average (tavg). Precipitation variables are seasonality (psea) and annual total (prec). Source data

Optimal regions for banana production were estimated using the *R*_90_ for total precipitation, mean annual temperature and temperature seasonality, each adjusted for irrigation level. Other climatic variables were omitted either because they were not strongly restricted for banana in relation to other land-use classes or because they were highly correlated with other climatic variables (Extended Data Fig. 4). Elevation, soil pH, population density and distance to port were also included to define the optimal region for banana. We arbitrarily restricted the potential area for banana to grid cells with at least 1% crop cover to avoid largely undeveloped protected and wilderness areas.

**Fig. 4:**
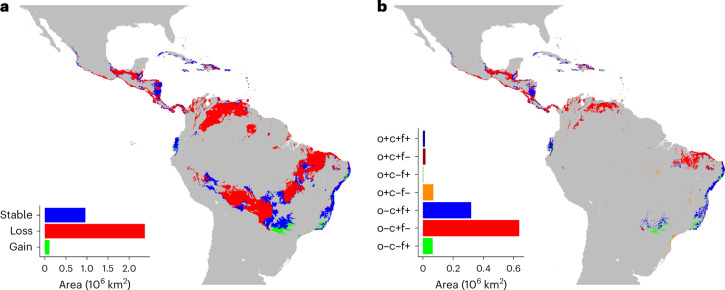
Suitability for export banana production. **a**, Suitability based on climatic and edaphic factors. The green areas are currently unsuitable (suboptimal) but will become suitable (optimal) in the future; the red areas are currently suitable but will become unsuitable. The blue areas will remain suitable. **b**, Observed distribution and suitability based on climatic, edaphic and socio-economic factors. Grid cells are classified by observed banana presence (o+) or absence (o−), predicted current suitability (c+) or unsuitability (c−) and predicted future suitability (f+) or unsuitability (f−). The inset shows the total area of each category, except the o−c−f− category (grey). Source data

**Extended Data Fig. 5:**
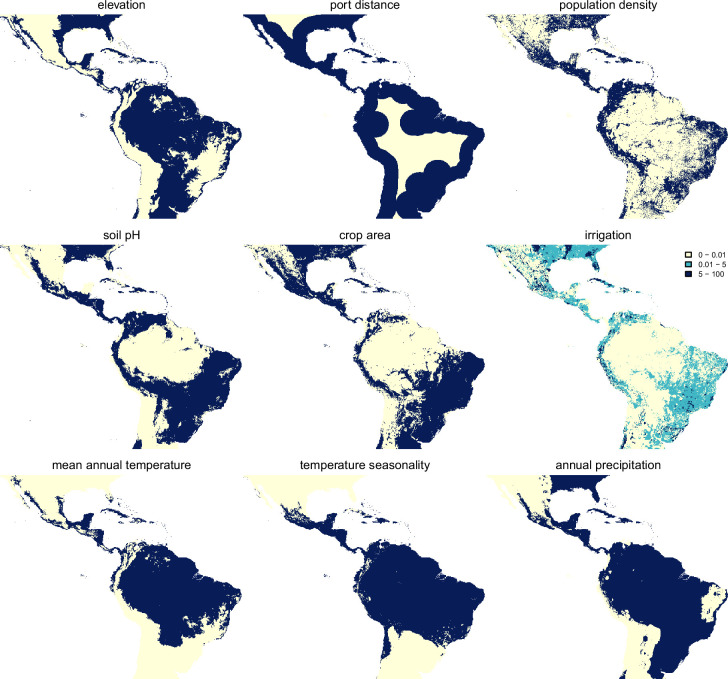
Current suitability for banana. Binary images show suitability (dark blue) for banana based on R_90_ for edaphic, socioeconomic and climatic predictors. Climatic predictors are adjusted for low ( < 5 %) and high (≥ 5 %) irrigation. Irrigation (% area) is shown categorized. Source data

Considering climatic and edaphic factors (elevation and soil pH), Central America, the northern and southern borders of the Amazon basin, and coastal Brazil are currently the most suitable (that is, optimal) for banana production (Fig. 4a and Extended Data Fig. 5). Banana plantations currently occupy only 9.0% of the area (on the basis of five-arcminute grid cells) that is predicted to be suitable under the recent historical climate (Fig. 4b and Supplementary Table 6). Both the total suitable area and the current production area that is suitable shrink dramatically (by 60% overall) under the SSP2-4.5 climate change scenario when considering both climatic and socio-economic constraints (Fig. 4b). In particular, Colombia and Venezuela are predicted to become almost entirely suboptimal for export production. Ecuador, a major exporter, is the only nation to see a small increase in suitable area and to maintain suitability for areas that currently have banana production. Suitable area is maintained primarily on the Atlantic coast of Brazil, with smaller areas in Nicaragua, Bolivia, Ecuador and Mexico (blue in Fig. 4b). Southern Brazil (the states of Mato Grosso do Sul, São Paulo, Paraná and Rio de Janeiro) and parts of the Atlantic coast are the only regions that are likely to become substantially more suitable for production in the future (green in Fig. 4b). Banana was detected in some areas, mainly in central Brazil, deemed to be unsuitable (orange in Fig. 4b). Overall, the suitable area considering only current climatic and edaphic factors (pH and elevation) is 3.34 × 10^6^ km^2^, reduced to 0.99 × 10^6^ km^2^ when population density, distance to port and crop cover are considered. Under future climate and edaphic constraints, the suitable area is 1.07 × 10^6^ km^2^, reduced to 0.40 × 10^6^ km^2^ when socio-economic factors are included (Supplementary Table 7).

**Fig. 5:**
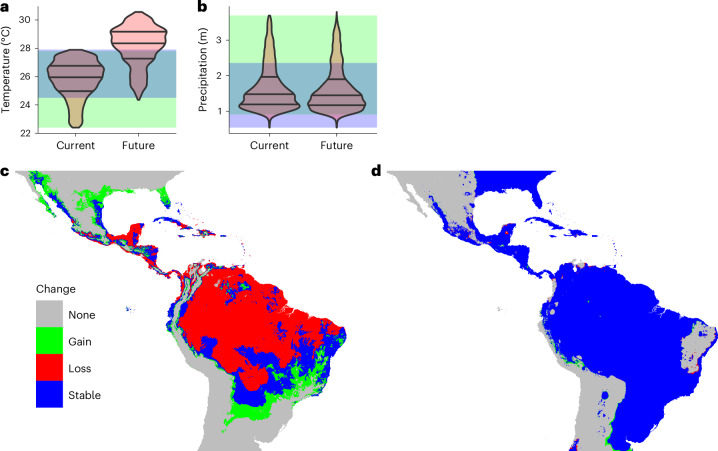
Temperature increase and suitable area. **a**,**b**, Area-weighted density of mean annual temperature (**a**) and annual precipitation (**b**) for currently suitable regions now and under future climate conditions (pink). The grey horizontal lines indicate the median and interquartile ranges. The distribution of suitable areas is compared with the *R*_90_ for low-irrigation areas (green), high-irrigation areas (blue) and their overlap (teal). **c**,**d**, Changes in suitable areas considering projected temperature change alone (**c**) and projected precipitation change alone (**d**). Source data

**Extended Data Fig. 6:**
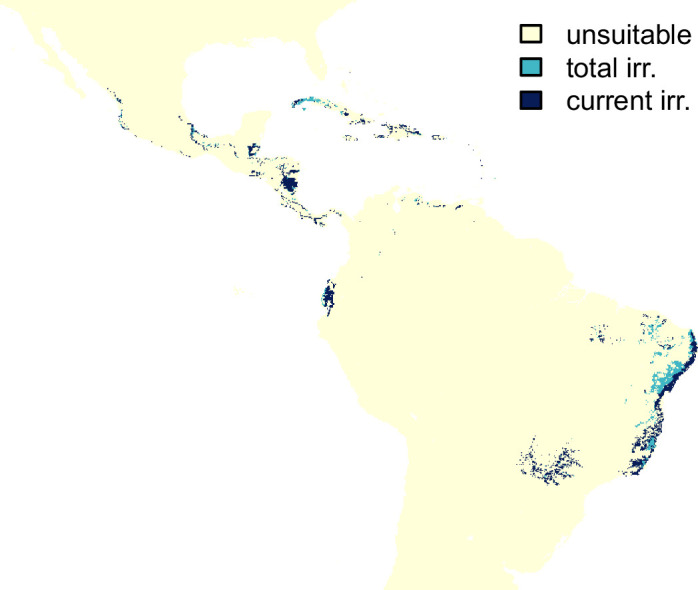
Future suitability for banana, by irrigation provision. Yellow denotes areas unsuitable for banana based on R_90_ for edaphic, socioeconomic and climatic predictors. Dark blue denotes areas suitable for banana with mean annual temperature, temperature seasonality and annual precipitation adjusted for current levels of irrigation provision. Green denotes additional suitable areas if irrigation provision were extended to the entire region. Source data

**Extended Data Fig. 7:**
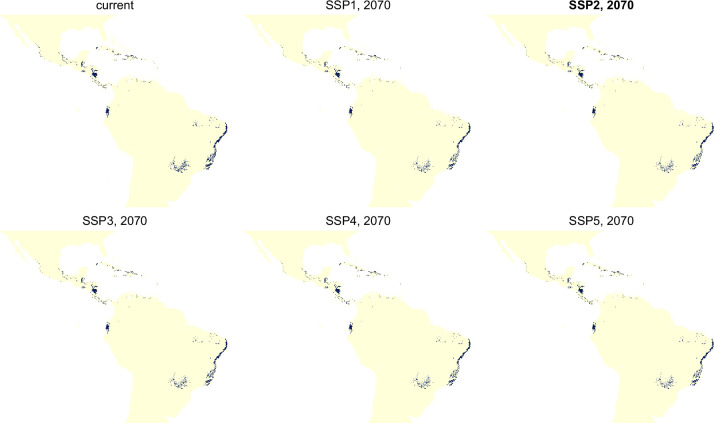
Future suitability for banana under different population density scenarios. Binary images show suitability (dark blue) for banana based on R_90_ for edaphic, socioeconomic and and future (SSP2-4.5, 2061–2080) climatic predictors. Climatic predictors are adjusted for low ( < 5 %) and high (≥ 5 %) irrigation. Panels show suitability based on current (2020) population density (top left) and future SSP population density projections for 2070. Source data

**Extended Data Fig. 8:**
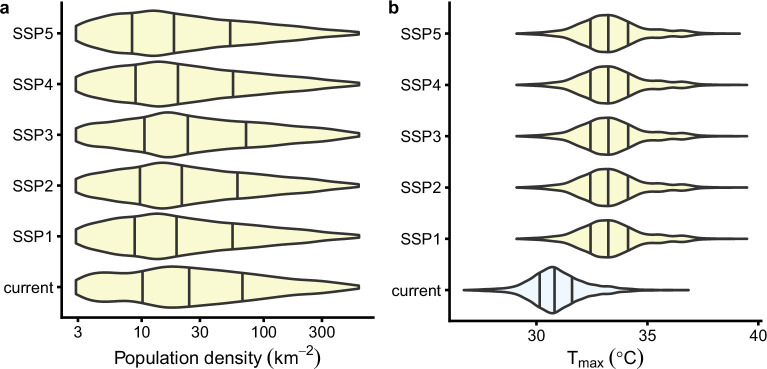
Population density and maximum temperature of warmest month. **a**) Violin plots show population density for grid cells most suitable for banana production using R_90_ limits for socioeconomic (port distance, crop cover) and edaphic factors (elevation, soil pH), future climate (SSP2-4.5, 2061-2080, adjusted by irrigation level) and different population density projections (current population in 2020, and SSP1–SSP5 scenarios for 2070). Population density is log-transformed for clarity. Vertical lines within violin plots show median and interquartile range. **b**) Maximum temperature of warmest month (T_max_) for suitable areas for banana production under ‘current’ climate (1970–2000, pale blue fill) and population density, and future climate (SSP2-4.5, 2061–2080, yellow fill) and population densities under different SSPs. Source data

Increasing temperature is the sole climatic driver of suitable area loss (Fig. 5). Considering the current suitable area, the distribution of mean annual temperature lies within the *R*_90_ for low- and high-irrigation areas (by definition). Under the future climate scenario, much of the currently suitable area becomes too warm, with mean annual temperatures exceeding 30 °C in some areas. In contrast, the annual precipitation distribution for currently suitable areas does not change noticeably. The establishment of irrigation where required across the region could expand the future suitable area by 45% compared with future climate under current irrigation, other factors being equal (Supplementary Table 8 and Extended Data Fig. 6). The total suitable area would shrink by 41% compared with current climate and irrigation provision, substantially less than the 60% loss under current irrigation. The dry Atlantic coast of Brazil would benefit the most in terms of absolute area, while Venezuela, Cuba and Mexico would benefit in relative terms. The trajectory of future population distributions under different SSPs has little influence on the area most suitable for banana (Extended Data Figs. 7 and 8a). However, the maximum temperature in the warmest month increases significantly (from mean 30.9 to 33.4 °C) under climate change (Extended Data Fig. 8b).

**Fig. 6:**
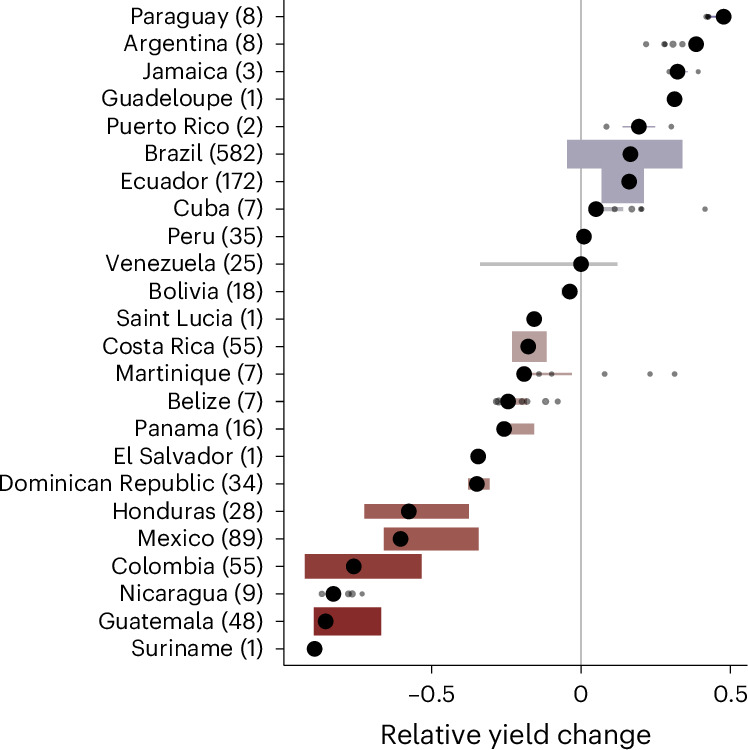
Projected relative yield change. The black points show the banana-area-weighted median climate-dependent yield change between recent historical and projected future (SSP2-4.5, 2061–2080) climate. The width of the coloured bars indicates the banana-area-weighted interquartile range; the height is proportional to the total banana area in BM19. The colours indicate median change. The number of grid cells per country is given in parentheses. Individual values are shown for countries with fewer than ten grid cells. Source data

Climate-dependent relative yield in current banana producing areas declined for most countries between historical and projected future climate (Fig. 6). Ecuador and Brazil are the only major producers expected to see yield increases in current banana production areas due to climate change.

## Discussion

We have presented a high-resolution map of intensive banana production for Latin America and the Caribbean, BM19, showing that this major export crop is produced within narrow climatic limits associated with high levels of irrigation and close to areas of high population density and shipping ports. Under the assumption of no future labour migration and no expansion of irrigation or transport infrastructure to ports, we found that the most suitable area for export banana production under climate change is strongly restricted. Substantial adaptation measures will be needed to facilitate production in future optimal production areas.

The classifier developed for BM19 showed high accuracy and precision. High SAR backscatter due to the physical structure of banana plants, and low variability in backscatter over time as a consequence of intensive-production practices, enabled discrimination of banana plantations from other vegetation types. However, other landscape features, particularly bare slopes in hilly areas, could potentially introduce classification errors. Such artefacts were largely corrected for by augmenting the SAR data with multi-spectral (Normalized Difference Vegetation Index, NDVI) and topographical (slope) information. As in previous studies^25^, we were unable to distinguish banana or plantain grown in less intensive or mixed cropping systems, which are common in Haiti (T. Lescot, personal communication) and other Caribbean islands.

Our classifier relied on data from the ESA Sentinel missions. Training of a random forest classifier for Sentinel-1 SAR data using the LUCAS 2018 land-use survey enabled identification of 18 crop classes (and three non-crop classes) across Europe at 10-m resolution, though with variable accuracy^27^. These classifiers benefited from intensive ground-truth surveys, with LUCAS contributing 87,853 data points to the European Union crop map. Such extensive ground-truth information is not currently available for Latin America, although field data have been used to develop distribution maps for some other crops such as soybean^28^. The stature and distinctive hexagonal planting arrangement of commercial oil palm plantations have facilitated mapping of this export crop by enabling training datasets to be developed by inspection of high-resolution aerial imagery^29^. The high spatial resolution (minimum area 0.5 ha) obtainable using the Sentinel data has enabled detailed analysis of spatio-temporal dynamics—for example, the loss and recovery of banana plantations after extreme weather events^24^. However, in the present study, we aggregated the banana distribution to match other spatial datasets required for our analyses.

We found that the BM19 distribution was closer to that predicted by the SPAM algorithm^30^ than by GAEZ^31^. SPAM downscales official crop production statistics for national or subnational administrative units to gridded cropland datasets using GAEZ crop suitability estimates and gridded estimates of irrigation provision. GAEZ uses similar datasets to SPAM, but the algorithm results in substantially different spatial crop allocation^32^. GAEZ models agroclimatic suitability for crops using a combination of climatic and edaphic tolerances. For banana, optimal conditions occur when more than one third of the growing season has a mean temperature of 20–30 °C, less than half the growing season between 10 and 15 °C, and no periods above 30 °C or below 10 °C. Suboptimal conditions allow temperatures above 30 °C for up to one sixth of the growing season, but no periods below 10 °C. Though not directly comparable, our *R*_90_ for mean annual temperature (22.9–27.9 °C without considering irrigation) is consistent with the GAEZ temperature profile. GAEZ models the effect of water availability on yield by comparing actual crop evapotranspiration with maximum crop evapotranspiration; hence, the relationship with precipitation is indirect. The approximate range of seasonal evapotranspiration for banana is estimated as 700–1,700 mm (ref. ^33^), which spans the 5th percentile for low-irrigation banana area in BM19 (953 mm), suggesting a sufficient level of rainfall to maximize yields across most of the distribution. We found that total precipitation could be lower, and precipitation seasonality could be greater, in high-irrigation areas, confirming that irrigation is associated with a wider climatic niche for this crop.

Our models suggest that future climate change will severely restrict the area most suitable for export banana production, particularly due to rising temperature. Temperature change was also found to be the dominant driver of yield trends in a historical analysis of global banana production^20^. In contrast, changing precipitation has less influence on future suitability as the precipitation range for banana is relatively wide. Investment in irrigation could extend the suitable area substantially, particularly in Brazil, where the area equipped for irrigation has increased greatly (though sustainably) in recent years^34^. A potential limitation in our analysis is the reliability of CMIP6 global circulation model precipitation projections, which in certain regions of South America tend to overestimate the frequency of dry periods relative to observations^35^. The absence of a strong effect of precipitation is partly due to the presence of irrigation; hence, the maintenance of irrigation water supplies in the future will be key, along with breeding of drought-tolerant banana varieties^36^. Currently, the banana industry is fighting a devastating fungal pathogen, *Fusarium* wilt Tropical Race 4 (caused by the fungus *Fusarium oxysporum* f.sp. *cubense*), which was first reported in Colombia in 2019 and has since been detected in Peru^37^. Efforts are underway to develop Cavendish cultivars resistant to this disease^38^, but our results suggest that climate change will lead to extreme temperatures that will severely reduce suitable production areas and yields in many important exporting countries and expose banana workers to greater risk of heat stress, with negative impacts on health and productivity^39,40^. The reduction and redistribution of highly productive areas across Central America, Latin America and the Caribbean would require the global banana value chain to adapt. This could stimulate a larger reorganization of international trade flows, with as yet unexplored consequences for local agricultural economies in producer countries to maintain demands for nutritional diversity in non-producing countries.

## Methods

Except where specified, all analyses were performed using Google Earth Engine (https://earthengine.google.com/) and R^41^ v.4.3.2. R package terra^42^ v.1.7-55 was used for raster data manipulation. Vectors of countries were obtained from the Database of Global Administrative Areas (GADM) using R package geodata^43^ v.0.5-9. The figures were made using R packages ggplot2 (ref. ^44^) v.3.5.1, tidyterra^45^ v. 0.7.0, ggspatial^46^ v.1.1.9 and ggcorplot^47^ v.0.1.4.1.

Our ROI includes North America, Central America, South America and the Caribbean islands between the latitudes 35° N and 37° S. The data sources used in the analysis are given in Supplementary Table 1.

### Ground-truth data

We sourced polygons of banana plantations that were active in 2019 from the Dominican Republic (source: BANELINO banana producers association, Santa Cruz de Mao, Dominican Republic) and Costa Rica (source: CORBANA national banana corporation, San José, Costa Rica). These polygons were digitized from ground surveys and represent a subset of the active plantation area in each country. In addition, a digitized paper map of banana plantation area for Belize (valid for 2012–2014) (source: Banana Growers Association, Big Creek, Belize) was updated using visual interpretation of high-resolution aerial imagery for 2019 available from Google Earth’s timeline feature. Large banana plantations are visually distinguishable from the surrounding landscape in such high-resolution images^21^. Hence, we have high confidence that these modified polygons represent active plantations in Belize for 2019. Preliminary runs of our classification method (detailed below) suggested some misclassification of banana plantations as non-banana plantations, which were most apparent in southern Brazil. We therefore augmented the banana plantation training data from this region. Using training polygons from the Dominican Republic, Costa Rica and Belize, we identified the characteristic distribution of Sentinel-1 vertical polarization (VV) backscatter values (median annual value for 2019) for plantations. We widened the bounds of this distribution and used it as a threshold (for the median annual Sentinel-1 VV backscatter for 2019) to generate a binary image of southern Brazil. This binary image was overlaid with high-resolution aerial imagery for 2019 available from Google Earth to manually digitize additional plantation polygons in the region. In total, the banana ground-truth dataset contained 156 polygons spread over Belize, the Dominican Republic, Costa Rica and Brazil.

In addition to the banana plantation polygons, we included five other land-cover classes in our classification process. These were generic classes of forest, undulating terrain with sparse vegetation, built-up areas, other crops and mangroves. The choice of including these generic classes was through an iterative trial-and-error process of preliminary classification runs and identifying the potential land-cover types that reduced the accuracy of the banana plantation class (primarily through false positives). For example, plants in commercial banana plantations tend to show high backscatter values in SAR imagery due to their large leaves and upright stature strongly reflecting the incident SAR signal. As commercial plantations operate on a continuous production schedule (to meet weekly export schedules), a low variation in backscatter values can be expected over time (that is, a weak seasonal signature). However, these characteristics are logically also shared by buildings and ridges, which can reflect the incident SAR signal to a large extent, and the intensity of the reflection would vary little over time. Training polygons for these generic classes were also digitized using high-resolution aerial imagery for 2019 available through Google Earth’s timeline feature.

### Mapping of banana plantations

Classification and mapping of banana plantations was carried out in Google Earth Engine. The study region was divided into 2° × 2° tiles in Earth Engine to facilitate efficient data processing and storage. We created an image collection for all Sentinel-1 and Sentinel-2 images for 2019 that covered the study region. The Sentinel-1 image collection was used to derive four data layers—annual median VV backscatter, annual median VH backscatter, annual VV standard deviation and annual VH standard deviation. Images in the Sentinel-2 image collection were first passed through a function that masks cloud-contaminated pixels using each image’s QA60 band. Band 4 and band 8 were then used to derive an annual median NDVI layer. A slope layer was derived using the 90-m-resolution Shuttle Radar Topography Mission Digital Elevation Model. Slope was included in the classification as commercial banana cultivation is carried out on relatively flat terrain. These six derived layers were concatenated or stacked and formed the dataset (henceforth referred to as the data stack) to build a classifier and thence to map plantations over the study extent.

Random sampling points were generated within the polygons of the six training classes. Values from the data stack were extracted to these sampling points. This represents the training dataset. The number of points generated within each class were *n* = 1,000 for banana, *n* = 1,000 for forest, *n* = 1,000 for undulating terrain with sparse vegetation, *n* = 100 for built-up areas, *n* = 200 for other crops and *n* = 200 for mangroves. The differences in the number of sampling points for each class reflect the severity of banana plantation classification inaccuracies that the respective generic land-cover classes generated, as well as the memory restrictions imposed by Google Earth Engine on the size of the training dataset. We used the random forest algorithm with 50 decision trees to build the classifier, which we then used to predict the presence of banana plantation pixels across the study extent. To reduce fine-scale artefacts of the classification, we enumerated the contiguity of the classified banana pixels and only retained patches of pixels that were ≥50 pixels. As the native resolution of the classification was 10 m, mapped plantations were ≥0.5 ha. Our method is therefore not capable of mapping the production area of smallholders and should be used exclusively for mapping larger commercial plantations.

To evaluate the overall accuracy of our classification, we generated a fresh set of 500 points per land-cover class, to which we extracted values from the data stack and calculated a confusion matrix^48^ after running this validation dataset through the classifier. To assess banana-plantation-specific measures of classifier performance, we extracted values from the validation error matrix and calculated the following:

$${\rm{Accuracy}}=({\rm{TP}}+{\rm{TN}})/({\rm{TP}}+{\rm{FP}}+{\rm{FN}}+{\rm{TN}})$$

$${\rm{Precision}}=({\rm{TP}})/({\rm{TP}}+{\rm{FP}})$$

$${\rm{Recall}}=({\rm{TP}})/({\rm{TP}}+{\rm{FN}})$$

$${F}_{1}=2{\times }(({\rm{precision}}{\times }{\rm{recall}})/({\rm{precision}}+{\rm{recall}}))$$

where TP are true positives, TN are true negatives, FP are false positives and FN are false negatives.

### Comparison of BM19 with existing crop distribution estimates and production statistics

Total BM19 plantation areas by country were compared with available FAO harvested area data for the categories ‘banana’ and ‘plantain and cooking bananas’ for the year 2019. BM19 aggregated to a five-arcminute grid was compared with harvested area in SPAM 2010 v.2.0 (ref. ^49^) and the GAEZ+ 2015 harvested area estimate^50^.

### Banana plantation occupancy of climatic, edaphic and socio-economic space

We employed bioclimatic (rectilinear surface range) envelope modelling^51^ to obtain the *R*_90_ for a set of climatic and socio-economic variables that we hypothesized would influence the selection of areas for banana production. Our predictors (detailed below) were bioclimatic variables, elevation, area equipped for irrigation, latitude, soil pH, human population density and distance to shipping port.

The rectilinear surface range method simply calculates the lower and upper bounds of a variable within which a certain fraction (here, 90%) of the area of a species is found. We interpret the *R*_90_ envelopes as those regions most desirable or suitable for export banana production, rather than the conditions under which banana could be grown. Hence, we refer to ‘optimal’ or ‘suitable’ areas while recognizing that banana can be grown under a wider range of conditions. BM19 and the predictors were aggregated to five-arcminute resolution where necessary, and *R*_90_ was estimated by weighting grid cells by banana area. *R*_90_ values for BM19 banana area were compared with *R*_90_ for all cropland and *R*_90_ for the ROI land surface, to determine which predictors were restrictive to the observed distribution of banana. Cropland extent was obtained from the Global Land and Discovery project^52^ at 0.025° resolution.

Recent historical (1970 to 2000) bioclimatic variables were obtained from WorldClim at five-arcminute resolution^53^. To minimize redundancy among climatic predictors, correlations among bioclimatic variables in WorldClim were used to identify a suitable subset: mean annual temperature (bioclimatic variable BIO1), mean diurnal temperature range (BIO2), temperature seasonality (BIO4) and annual precipitation (BIO12). This subset was used to predict the region most suitable for banana production under recent historical and future climate (see below).

Irrigation is used to extend the range of crop species into chronically and seasonally drier, as well as potentially hotter, areas than would be possible under rainfed agriculture alone. We estimated *R*_90_ for selected bioclimatic predictors in two groups determined by area equipped for irrigation^54^: low-irrigation production with irrigation area of 0–5% and high-irrigation production with irrigation >5%. The 5% boundary between low- and high-irrigation areas was determined by the median area equipped for irrigation in banana-producing grid cells. Little published information is available regarding optimal soil properties for banana production, though in Australia banana tends to be grown in slightly acidic soils^55^. We therefore estimated *R*_90_ for soil pH at 0–5 cm using SoilGrids250m data^56^. Given that Cavendish bananas are exported by sea, we included distance from the centre of each grid cell to the nearest international port listed in the World Port Index^57^. Banana production and harvesting rely on manual labour, so we estimated *R*_90_ for population density^58^.

### Projecting future optimal banana plantation distribution

For the future climate scenario, we used ensemble averages of 12 CMIP6 global circulation model outputs driven by SSP2-4.5 for the time period 2061–2080. These global circulation model outputs were obtained from WorldClim at five-arcminute resolution^53^. The chosen SSP sees emissions peak in 2040 and then gradually decline, limiting global warming to under 3 °C by 2100. The region most suitable for banana production was estimated from *R*_90_ values using future mean annual temperature, temperature seasonality and annual precipitation, holding irrigation, cropland, soil pH, population density and distance to port at the current values.

The area equipped for irrigation has grown in recent decades^34^ and could continue to increase in the future. We estimated the maximum future extent of banana growing areas by widening the *R*_90_ for mean annual temperature, temperature seasonality and annual precipitation to incorporate the effect of irrigation. For example, *R*_90_ for annual precipitation is 912–3,690 mm for low-irrigation (<5%) areas and 543–2,356 mm for high-irrigation (≥5%) areas (Supplementary Table 5). To estimate future suitable area with unrestricted irrigation, the *R*_90_ for precipitation was thus extended to 543–3,690 mm.

Population densities are likely to change in the future due to population growth and trends such as urbanization^59^. The SSPs provide a range of scenarios for changes in population size and distribution^59^. For climate change, we chose SSP2, known as ‘Middle of the road’. Demographic change in SSP2 is characterized by medium levels of population growth and medium rates of urbanization, with spatial distributions following historical patterns. Population growth rates in high-fertility, low-fertility and rich low-fertility countries, along with urbanization levels in low-, medium- and high-income countries, vary among the other SSPs: SSP1 (Sustainability), SSP3 (Regional rivalry), SSP4 (Inequality) and SSP5 (Fossil-fuelled development). We explored the effect of projected population density in 2070 among SSPs on the area most suitable for banana using downscaled gridded population density projections^60^, assuming that the dependence of banana production on labour does not change in the future. Exposure to high temperatures is a serious health risk for agricultural labourers^39,40^. We calculated the maximum temperature of the warmest month (BIO5) for suitable areas under the current climate and compared this to the maximum temperatures of suitable areas under SSP2-4.5 climate and population distributions under SSP1–SSP5.

### Relative yield estimation

We estimated climate-dependent relative yield (*Y*_C_) for all five-arcminute grid cells containing banana (BM19) using response curves estimated from national and subnational production data for the Latin America and the Caribbean region^20^. Actual yield is a function of *Y*_C_ and other factors such as agrochemical inputs and management, which vary among countries and over time^20^. *Y*_C_ was estimated as the product of temperature-dependent (*Y*_T_) and precipitation-dependent (*Y*_P_) yields:

$${Y}_{{\mathrm{C}}}={Y}_{{\mathrm{T}}}{\times }{Y}_{{\mathrm{P}}}$$

*Y*_T_ and *Y*_P_ were estimated using beta functions that employ minimum (min), optimum (opt) and maximum (max) values of mean annual temperature (*T*) and annual precipitation (*P*) for yield. For example, the beta function for temperature is:

$${Y}_{{\mathrm{T}}}=\left(\frac{{T}_{\max }-T}{{T}_{\max }-{T}_{{\mathrm{opt}}}}\right){\left(\frac{T-{T}_{\min }}{{T}_{{\mathrm{opt}}}-{T}_{\min }}\right)}^{\frac{\left({T}_{{\mathrm{opt}}}-{T}_{\min }\right)}{\left({T}_{\max }-{T}_{{\mathrm{opt}}}\right)}}$$

*Y*_C_, *Y*_T_ and *Y*_P_ take values of one under optimal conditions, and zero if any predictor falls below the minimum or above the maximum. For Latin America and the Caribbean, the cardinal (minimum, optimum and maximum) values for temperature are 20.0, 26.8 and 29.4 °C. The values for precipitation are 85.5, 2,646 and 5,307 mm. *Y*_C_ was estimated for current (*Y*_Cc_) and future (*Y*_Cf_) conditions using recent historical (1970–2000) and projected (multimodel average, SSP2-4.5, 2061–2080) climates. Change in yield per country was estimated as the banana-area-weighted median and interquartile range of the relative difference between current and future relative yield (*Y*_Cf_ − *Y*_Cc_).

### Reporting summary

Further information on research design is available in the Nature Portfolio Reporting Summary linked to this article.

## Supplementary information

Supplementary Information Supplementary Tables 1–8.

Reporting Summary

## Source data

Source Data Fig. 1 GeoTIFF image files.

Source Data Fig. 2 Statistical source data (CSV).

Source Data Fig. 3 Statistical source data (CSV).

Source Data Fig. 4 GeoTIFF image file and category table (CSV).

Source Data Fig. 5 GeoTIFF image file and category table (CSV).

Source Data Fig. 6 GeoTIFF image file and category table (CSV).

Source Data Extended Data Fig. 1 Statistical source data (CSV).

Source Data Extended Data Fig. 4 Statistical source data (CSV).

Source Data Extended Data Fig. 5 GeoTIFF image files.

Source Data Extended Data Fig. 6 GeoTIFF image file and category table (CSV).

Source Data Extended Data Fig. 7 GeoTIFF image files.

Source Data Extended Data Fig. 8 Statistical source data (CSV).

## Data Availability

All data used for analysis are available from the sources listed in Supplementary Table 1, except the banana production locations supplied by BANELINO (Santa Cruz de Mao, Dominican Republic), CORBANA (San José, Costa Rica) and the Banana Growers Association (Big Creek, Belize). The data are available on request from these organizations. The BM19 banana production map tiles are available via figshare at 10.6084/m9.figshare.26509024 (ref. ^61^) as TIF files. Source data are provided with this paper.

## Code availability

A land-use classifier was developed in JavaScript for Google Earth Engine. The code is available at https://code.earthengine.google.com/dd3aa4e7547fd21a1c16a8f03d3d74ff?noload=true and https://code.earthengine.google.com/5da98b1455951c36dcc133c48589efa6?noload=true.
