# Supplementary material for: Socio-economic factors constrain climate change adaptation in a tropical export crop
Source: Nat Food. 2025 Mar 6;6(4):343–52. doi: 10.1038/s43016-025-01130-1 (PMC12018255; doi:10.1038/s43016-025-01130-1)
Supplement: Supplementary file 1 — Supplementary Tables 1–8. [file 43016_2025_1130_MOESM1_ESM.pdf]

nature food

Supplementary information

https://doi.org/10.1038/s43016-025-01130-1

Socio-economic factors constrain climate change adaptation in a tropical export crop

In the format provided by the authors and unedited

Supplementary Information

Supplementary Tables 1–8.

Supplementary Table 1. Data sources.

Dataset	URL
Area equipped for irrigation, gridded (FAO AQUASTAT)	https://data.apps.fao.org/catalog/iso/a6fe7f5b-b887-452b-922a-668b7771b450
Bioclimatic variables (future SSP2–4.5 mean, 2061-2080)	https://worldclim.org
Bioclimatic variables (recent historical mean, 1970–2000)	https://worldclim.org
Country border vectors	https://gadm.org
Cropland distribution (GLAD)	https://glad.umd.edu/dataset/croplands
ESA Copernicus Sentinel 1 SAR	https://earthengine.google.com/
ESA Copernicus Sentinel 2 multispectral	https://earthengine.google.com/
Harvested area of banana and plantain by country	https://www.fao.org/faostat/
Harvested area of banana, gridded (GAEZ)	https://gaez-data-portal-hqfao.hub.arcgis.com/
Harvested area of banana, gridded (SPAM)	https://mapspam.info/
Population density in 2020, gridded (GPWv4)	http://sedac.ciesin.columbia.edu/data/collection/gpw-v4/
Population density projections under shared socioeconomic pathways	https://doi.org/10.6084/ m9.figshare.19608594.v2
Shipping port locations (WPI)	https://msi.nga.mil/Publications/WPI
Soil pH, 0-5cm depth, gridded (SoilGRIDS)	https://www.isric.org/explore/soilgrids

Supplementary Table 2. Confusion matrix for the BM19 classifier. Classifier performance for the banana class was: accuracy 0.99, precision 0.96, sensitivity 1.00, specificity 0.99 and F_1 score 0.98.

	Predicted						
Observed	Banana	Forest	Terrain	Built	Other crop	Mangrove	Total
Banana	500	0	0	0	0	0	500
Forest	1	496	3	0	0	0	500
Terrain	0	5	495	0	0	0	500
Built	2	28	35	407	27	1	500
Other crop	0	78	58	2	360	2	500
Mangrove	17	196	39	2	5	241	500
Total pred	520	803	630	411	392	244	3000

Supplementary Table 3. Banana area by country (2019). Countries reporting banana statistics in FAOSTAT are included. Code refers to the labels used in Fig. S2. BM19 is the area detected by our classifier. FAOSTAT areas are given for categories ‘bananas’ and ‘plantains and cooking bananas’. FAOSTAT export is for bananas.

Country	Code	BM19 (ha)	FAOSTAT 2019			
			Banana (ha)	Plantain etc. (ha)	Total (ha)	Export (tonnes)
Antigua and Barbuda	Ant	0	39	0	39	0
Argentina	Arg	1366.7	8406	0	8406	0
Bahamas	Bah	0	416	25	441	0
Barbados	Bar	0	178	0	178	0
Belize	Bel	2166.7	3036	394	3430	85930
Bolivia	Bol	3872.6	20103	41501	61604	109349
Brazil	Bra	44890.8	462026	0	462026	78944
Colombia	Col	33898.2	105609	250575	356184	1896091
Costa Rica	Cos	53152.7	51622	6334	57956	2382317
Cuba	Cub	803.8	69949	0	69949	0
Curaçao	Cur	0	69949	0	69949	0
Dominica	Dom	0	2770	723	3493	336
Dominican Republic	DoR	7167.1	28861	48977	77838	393516
Ecuador	Ecu	102091	183347	135813	319160	6667584
El Salvador	ElS	13.8	966	1881	2847	0
Grenada	Gre	0	226	0	226	2
Guatemala	Gut	47479.4	69264	8738	78002	2585781
Guyana	Guy	14.6	571	3226	3797	3
Haiti	Hai	0	58167	38194	96361	0
Honduras	Hon	11983.5	14303	7833	22136	593401
Jamaica	Jam	25.7	8376	2383	10759	647
Mexico	Mex	34902.7	79593	0	79593	571919
Nicaragua	Nic	2315.1	1867	15891	17758	140441
Panama	Pan	10224.8	7000	17627	24627	611832
Paraguay	Par	170.4	8738	0	8738	66730
Peru	Per	3238.6	175407	0	175407	221823
Puerto Rico	Pue	89.6	1551	3445	4996	0
Saint Lucia	Slu	18.6	357	137	494	10162
Saint Vincent and the Grenadines	SVG	0	6117	154	6271	2512
Suriname	Sur	48.9	1051	471	1522	6091
Trinidad and Tobago	TTo	0	1026	1498	2524	16
Venezuela	Ven	715.3	39196	64258	103454	95
TOTAL		360662.5	1480087	650078	2130165	16425522

Supplementary Table 4. Banana distribution in relation to climatic, edaphic and socioeconomic variables. All variables were standardized to 5 arc minute resolution. Code refers to the labels in Fig. 2. Land use refers to BM19 banana plantation estimates, weighted by banana area per grid cell. Crops refers to GLAD cropland area. All refers to the entire land surface in the ROI. Q values are quantiles (5th to 95th percentile) of the area of land use for each variable. The 90th percentile range (R_{90}) is (Q_{05}–Q_{95}).

Variable	Code	Land use	Q_{05}	Q_{25}	Q_{50}	Q_{75}	Q_{95}
Elevation (m)	ele	Banana	7	13	28	82	439
		Crops	24	110	323	595	1608
		All	23	115	260	673	2683
Irrigation (%)	irr	Banana	0.0	0.0	4.9	23.5	65.3
		Crops	0.0	0.0	0.2	1.5	26.0
		All	0.0	0.0	0.0	0.2	6.3
Latitude (˚)	lat	Banana	-18.0	-2.8	9.4	14.0	18.7
		Crops	-34.9	-25.6	-15.4	12.1	33.0
		All	-31.7	-17.4	-6.0	8.9	32.7
Soil pH (0–5cm)	ph	Banana	5.0	5.5	5.9	6.2	6.7
		Crops	5.0	5.4	5.9	6.6	7.5
		All	4.1	4.9	5.5	6.5	7.9
Population (km^{-2})	pop	Banana	2.9	52.4	85.9	166.8	606.4
		Crops	0.1	0.9	3.7	20.7	167.3
		All	0.0	0.2	1.3	8.5	104.2
Port Distance (km)	port	Banana	14.3	30.7	50.5	85.5	518.3
		Crops	45.5	142.4	288.2	461.1	871.6
		All	48.9	162.5	322.3	534.9	877.7
Precipitation Annual (mm)	prec	Banana	566.0	1063.8	1778.5	2495.5	3645.3
		Crops	482.0	832.0	1195.0	1538.0	2001.0
		All	218.0	812.0	1411.0	1999.0	2873.0
Precipitation Seasonality (%)	psea	Banana	29.2	42.3	83.3	95.3	115.4
		Crops	15.9	43.2	63.9	82.8	108.0
		All	17.0	42.8	61.2	79.2	108.3
Temperature Mean (˚C)	tavg	Banana	22.9	25.2	26.1	26.9	27.9
		Crops	15.2	17.6	21.6	24.2	26.4
		All	11.1	18.6	23.6	25.8	26.9
Temperature Diurnal range (˚C)	tdir	Banana	7.7	8.5	9.6	11.4	13.1
		Crops	9.1	11.4	12.4	13.4	16.3
		All	8.4	9.9	11.6	13.2	17.2
Temperature Isothermality		Banana	61.6	73.8	76.7	78.6	89.5
		Crops	40	48.9	62.9	69.4	77.6
		All	41.6	54.4	68.5	77.8	86.9
Temperature Maximum (˚C)	tmax	Banana	30.2	31.3	32.0	33.9	35.5
		Crops	27.1	30.2	31.6	33.4	35.6
		All	21.5	30.5	32.2	33.5	35.6
Temperature Minimum (˚C)	tmin	Banana	13.5	18.5	19.2	20.8	22.7
		Crops	-1.1	4.5	10.5	14.6	19.8
		All	-3.3	5.6	14.1	19.1	21.6
Temperature Seasonality (˚C)	tsea	Banana	26.2	72.9	84.1	111.6	226.0
		Crops	66.8	133.0	251.9	490.5	789.7
		All	34.6	59.3	152.4	394.3	751.0

Supplementary Table 5. Banana distribution in relation to mean, minimum and maximum annual temperature and annual precipitation by irrigation class. Q values are quantiles.

Variable	Irrigation	Q05	Q25	Q50	Q75	Q95
Temperature Mean (˚C)	< 5 %	22.4	25.4	26.3	26.6	27.8
	≥ 5 %	24.5	25.2	25.5	27.2	27.9
Temperature Minimum (˚C)	< 5 %	13.2	18.3	20.4	21.3	22.9
	≥ 5 %	16.3	18.5	19.0	19.4	21.2
Temperature Maximum (˚C)	< 5 %	29.9	31.3	31.9	33.0	35.4
	≥ 5 %	30.4	31.5	32.3	34.5	35.7
Temperature Seasonality (˚C)	< 5 %	23.2	67.2	78.1	155.0	296.0
	≥ 5 %	47.2	75.3	99.0	109.0	167.0
Precipitation Total (mm)	< 5 %	912	1673	2473	3436	3690
	≥ 5 %	543	802	1210	1817	2356
Precipitation Seasonality (%)	< 5 %	27.8	33.8	46.9	68.4	100.0
	≥ 5 %	51.3	84.7	92.7	110.1	117.3

Supplementary Table 6. Banana suitability by country, historical (1970–2000) vs. SSP2–4.5 (2061–2080). Suitability was estimated using 90th percentile range (R_{90}) of total precipitation, mean annual temperature and temperature seasonality, each adjusted for irrigation level (< 5 % or ≥ 5 % area), along with elevation, soil pH, population density, distance to port, and a minimum crop area of 1 % per 5 arc minute grid cell. Values are areas (km^2) of grid cells in each class. Grid cells were classified by observed presence (o+) or absence (o-) of banana plantations, current suitability (c+) or not (c-), and future suitability (f+) or not (f-). Observed is the area of grid cells within which we found banana, Current is the predicted currently suitable area, Future is the predicted future suitable area. Change is the percentage change in suitability between current and future climate. Countries are listed in order of total currently suitable area (Current), then by total area.

Country	o-c-f-	o-c-f+	o-c+f-	o-c+f+	o+c-f-	o+c-f+	o+c+f-	o+c+f+	Observed	Current	Future	Change (%)
Brazil	7998216	58204	196712	165690	45204	240	680	1882	48006	364964	226016	-38
Ecuador	216130	3161	0	19314	3075	1367	0	9397	13839	28711	33239	16
Mexico	1839758	719	76672	18622	3656	0	3600	0	7256	98894	19341	-80
Colombia	1074784	0	53824	595	3892	0	757	0	4649	55176	595	-99
Costa Rica	22585	421	16659	6318	757	0	3200	590	4547	26767	7329	-73
Guatemala	63978	82	33374	7712	745	0	3146	83	3974	44315	7877	-82
Peru	1283851	0	1612	0	2979	0	0	0	2979	1612	0	-100
Dominican Republic	26825	405	10041	7358	1052	0	1290	404	2746	19093	8167	-57
Honduras	89847	0	12468	6365	578	0	1732	0	2310	20565	6365	-69
Venezuela	754146	337	147973	4804	2029	0	84	0	2113	152861	5141	-97
Bolivia	1079603	0	165	0	1473	0	0	0	1473	165	0	-100
Panama	35676	0	26643	9133	592	0	675	169	1436	36620	9302	-75
Nicaragua	49853	0	34084	37861	167	0	584	0	751	72529	37861	-48
Argentina	1787486	0	0	0	630	0	0	0	630	0	0	NA
Paraguay	394128	316	2369	1740	622	0	0	0	622	4109	2056	-50
Martinique	83	0	83	83	248	0	166	165	579	497	248	-50
Belize	10292	0	6620	3929	0	0	574	0	574	11123	3929	-65
Cuba	90544	80	2323	13077	555	0	0	0	555	15400	13157	-15
Jamaica	6994	0	894	2359	244	0	0	0	244	3253	2359	-27
Puerto Rico	6179	81	163	2113	81	0	0	81	162	2357	2275	-3
Suriname	146544	0	0	0	85	0	0	0	85	0	0	NA
El Salvador	10049	83	5906	4569	0	0	83	0	83	10558	4652	-56
Saint Lucia	249	0	166	0	83	0	0	0	83	166	0	-100
Guadeloupe	493	0	739	82	82	0	0	0	82	821	82	-90
United States	2017493	0	0	0	0	0	0	0	0	0	0	NA
Chile	393498	0	0	0	0	0	0	0	0	0	0	NA
Guyana	209374	0	340	0	0	0	0	0	0	340	0	-100
Uruguay	176862	0	0	0	0	0	0	0	0	0	0	NA

French Guiana	82034	0	0	0	0	0	0	0	0	0	0	NA
Haiti	13929	404	4530	6800	0	0	0	0	0	11330	7204	-36
Bahamas	10702	0	77	77	0	0	0	0	0	154	77	-50
Trinidad and Tobago	3531	0	1598	84	0	0	0	0	0	1682	84	-95
Turks and Caicos Islands	557	0	0	0	0	0	0	0	0	0	0	NA
Curaçao	418	0	0	0	0	0	0	0	0	0	0	NA
Dominica	412	0	0	165	0	0	0	0	0	165	165	0
Antigua and Barbuda	409	0	82	0	0	0	0	0	0	82	0	-100
Bonaire, Saint Eustatius and Saba	334	0	0	0	0	0	0	0	0	0	0	NA
Virgin Islands, U.S.	163	0	0	0	0	0	0	0	0	0	0	NA
Cayman Islands	162	0	0	0	0	0	0	0	0	0	0	NA
Barbados	83	0	416	0	0	0	0	0	0	416	0	-100
Saint Vincent and the Grenadines	83	0	83	83	0	0	0	0	0	166	83	-50
Aruba	83	0	0	0	0	0	0	0	0	0	0	NA
Anguilla	81	0	0	0	0	0	0	0	0	0	0	NA
Sint Maarten	81	0	0	0	0	0	0	0	0	0	0	NA
Grenada	0	0	167	84	0	0	0	0	0	251	84	-67
Montserrat	0	0	0	82	0	0	0	0	0	82	82	0
Saint Kitts and Nevis	0	0	0	82	0	0	0	0	0	82	82	0
**Total**	**19898582**	**64293**	**636783**	**319181**	**68829**	**1607**	**16571**	**12771**	**99778**	**985306**	**397852**	**-60**

Supplementary Table 7. Climatic, edaphic and socioeconomic constraints on banana production.

First row is total land area of the ROI. Subsequent rows give optimal banana area subject to various constraints. Climate refers to temperature and precipitation (adjusted for current irrigation). Last row assumes irrigation is available everywhere. Edaphic constraints are soil pH and elevation. Socioeconomic constraints are population density, distance to port and areas which currently have at least 1 % crop cover.

Region	Area (10^6 km^2)	% of total
Total land area	21.10	100
Climate (historical) & edaphic	3.34	15.8
Climate (future) & edaphic	1.07	5.1
Socioeconomic	3.14	14.9
Climate (historical) & socioeconomic	0.99	4.7
Climate (future) & socioeconomic	0.40	1.9
Climate (future), full irrigation & socioeconomic	0.58	2.7

Supplementary Table 8. Future banana suitability under current and total irrigation provision.
Columns 2–4 show area (km^2) optimal for banana production under assumptions of historical (HC) or future (FC) climate, and current (CI) or total (TI) irrigation provision. Change (%) shows percentage change in optimal area considering both climate change and total irrigation implementation (column 4 vs 2). Difference (%) gives the difference in suitable area under future climate if total irrigation was implemented (column 4 vs 2).

Country	HC HI	FC HI	FC TI	Change (%) HC:FC, CI:TI	Difference (%) FC, CI:TI
Brazil	364964	226016	358987	-2	59
Nicaragua	72529	37861	41366	-43	9
Ecuador	28711	33239	38366	34	15
Mexico	98894	19341	27512	-72	42
Cuba	15400	13157	30885	101	135
Panama	36620	9302	10232	-72	10
Dominican Republic	19093	8167	9464	-50	16
Guatemala	44315	7877	9274	-79	18
Costa Rica	26767	7328	8590	-68	17
Haiti	11330	7204	7448	-34	3
Honduras	20565	6365	8018	-61	26
Venezuela	152861	5141	8507	-94	65
El Salvador	10558	4652	4735	-55	2
Belize	11123	3929	5486	-51	40
Jamaica	3253	2359	2522	-22	7
Puerto Rico	2357	2276	2357	0	4
Paraguay	4109	2056	2056	-50	0
Colombia	55176	595	766	-99	29
Martinique	497	248	248	-50	0
Dominica	165	165	165	0	0
Grenada	251	84	84	-67	0
Trinidad and Tobago	1682	84	84	-95	0
Saint Vincent and the Grenadines	166	83	83	-50	0
Guadeloupe	821	82	82	-90	0
Montserrat	82	82	82	0	0
Saint Kitts and Nevis	82	82	82	0	0
Bahamas	154	77	77	-50	0
Anguilla	0	0	0	NA	NA
Antigua and Barbuda	82	0	0	-100	NA
Argentina	0	0	0	NA	NA
Aruba	0	0	0	NA	NA
Barbados	416	0	83	-80	NA
Bolivia	165	0	0	-100	NA
Bonaire, Saint Eustatius and Saba	0	0	0	NA	NA
Cayman Islands	0	0	0	NA	NA
Chile	0	0	0	NA	NA
Curaçao	0	0	0	NA	NA
French Guiana	0	0	0	NA	NA
Guyana	340	0	0	-100	NA
Peru	1612	0	0	-100	NA
Saint Lucia	166	0	83	-50	NA
Sint Maarten	0	0	0	NA	NA
Suriname	0	0	0	NA	NA
Turks and Caicos Islands	0	0	0	NA	NA
United States	0	0	0	NA	NA
Uruguay	0	0	0	NA	NA
Virgin Islands, U.S.	0	0	0	NA	NA
**Total**	**985306**	**397852**	**577724**	**-41**	**45**
