# Supplementary material for: Socio-economic factors constrain climate change adaptation in a tropical export crop
Source: Nat Food. 2025 Mar 6;6(4):343–52. doi: 10.1038/s43016-025-01130-1 (PMC12018255; doi:10.1038/s43016-025-01130-1)
Supplement: Supplementary file 2 — Reporting Summary [file 43016_2025_1130_MOESM2_ESM.pdf]

nature portfolio

Corresponding author(s): Daniel Bebber

Last updated by author(s): Aug 7, 2024

Reporting Summary

Nature Portfolio wishes to improve the reproducibility of the work that we publish. This form provides structure for consistency and transparency in reporting. For further information on Nature Portfolio policies, see our Editorial Policies and the Editorial Policy Checklist.

Statistics

For all statistical analyses, confirm that the following items are present in the figure legend, table legend, main text, or Methods section.

n/a	Confirmed	
☐	☒	The exact sample size (*n*) for each experimental group/condition, given as a discrete number and unit of measurement
☒	☐	A statement on whether measurements were taken from distinct samples or whether the same sample was measured repeatedly
☒	☐	The statistical test(s) used AND whether they are one- or two-sided<br>*Only common tests should be described solely by name; describe more complex techniques in the Methods section.*
☐	☒	A description of all covariates tested
☒	☐	A description of any assumptions or corrections, such as tests of normality and adjustment for multiple comparisons
☐	☒	A full description of the statistical parameters including central tendency (e.g. means) or other basic estimates (e.g. regression coefficient) AND variation (e.g. standard deviation) or associated estimates of uncertainty (e.g. confidence intervals)
☒	☐	For null hypothesis testing, the test statistic (e.g. *F*, *t*, *r*) with confidence intervals, effect sizes, degrees of freedom and *P* value noted<br>*Give P values as exact values whenever suitable.*
☒	☐	For Bayesian analysis, information on the choice of priors and Markov chain Monte Carlo settings
☒	☐	For hierarchical and complex designs, identification of the appropriate level for tests and full reporting of outcomes
☐	☒	Estimates of effect sizes (e.g. Cohen's *d*, Pearson's *r*), indicating how they were calculated

Our web collection on statistics for biologists contains articles on many of the points above.

Software and code

Policy information about availability of computer code

Data collection	A land use classifier was developed in Javascript for Google Earth Engine. The code is available at https://code.earthengine.google.com/dd3aa4e7547fd21a1c16a8f03d3d74ff?noload=true and https://code.earthengine.google.com/5da98b1455951c36dcc133c48589efa6?noload=true
Data analysis	Data analysis other than for the land use classifier was conducted in R version 4.3.2. Package terra version 1.7-55 was used for raster data manipulation. Vectors of countries were obtained from the Database of Global Administrative Areas (GADM) using package geodata version 0.5-9.

For manuscripts utilizing custom algorithms or software that are central to the research but not yet described in published literature, software must be made available to editors and reviewers. We strongly encourage code deposition in a community repository (e.g. GitHub). See the Nature Portfolio guidelines for submitting code & software for further information.

Data

Policy information about availability of data

All manuscripts must include a data availability statement. This statement should provide the following information, where applicable:

- Accession codes, unique identifiers, or web links for publicly available datasets
- A description of any restrictions on data availability
- For clinical datasets or third party data, please ensure that the statement adheres to our policy

All data used for analysis are available from sources listed in Table S1, with the exception of banana production locations supplied by BANELINO (Santa Cruz de Mao, Dominican Republic), CORBANA (San José, Costa Rica) and the Banana Growers Association (Big Creek, Belize). The data are available on request from these organizations. The BM19 banana production map tiles are available at https://doi.org/10.6084/m9.figshare.26509024 as Tag Image File Format (TIF) files

Human research participants

Policy information about studies involving human research participants and Sex and Gender in Research.

Reporting on sex and gender	NA
Population characteristics	NA
Recruitment	NA
Ethics oversight	NA

Note that full information on the approval of the study protocol must also be provided in the manuscript.

Field-specific reporting

Please select the one below that is the best fit for your research. If you are not sure, read the appropriate sections before making your selection.

☐ Life sciences ☐ Behavioural & social sciences ☒ Ecological, evolutionary & environmental sciences

For a reference copy of the document with all sections, see nature.com/documents/nr-reporting-summary-flat.pdf

Ecological, evolutionary & environmental sciences study design

All studies must disclose on these points even when the disclosure is negative.

Study description	The study analysed remote sensing data to generate a map of banana production in Latin America and the Caribbean, and analysed the banana distribution in relation to associated climatic, edaphic and socioeconomic data.
Research sample	The banana map was generated at 0.5 ha resolution. This was aggregated to 5 arc minute resolution for comparison with other datasets.
Sampling strategy	The entire region was analysed. A representative sample of banana plantation locations (N = 156 polygons) were obtained in Belize, Dominican Republic and Costa Rica.
Data collection	The remote sensing data were analysed in Google Earth Engine.
Timing and spatial scale	Satellite images were obtained for the year 2019.
Data exclusions	None
Reproducibility	NA
Randomization	NA
Blinding	NA

Did the study involve field work? ☐ Yes ☒ No

Reporting for specific materials, systems and methods

We require information from authors about some types of materials, experimental systems and methods used in many studies. Here, indicate whether each material, system or method listed is relevant to your study. If you are not sure if a list item applies to your research, read the appropriate section before selecting a response.

Materials & experimental systems

n/a	Involved in the study
☒	☐ Antibodies
☒	☐ Eukaryotic cell lines
☒	☐ Palaeontology and archaeology
☒	☐ Animals and other organisms
☒	☐ Clinical data
☒	☐ Dual use research of concern

Methods

n/a	Involved in the study
☒	☐ ChIP-seq
☒	☐ Flow cytometry
☒	☐ MRI-based neuroimaging
