# Supplementary figures and images for: Socio-economic factors constrain climate change adaptation in a tropical export crop
Source: Nat Food. 2025 Mar 6;6(4):343–52. doi: 10.1038/s43016-025-01130-1 (PMC12018255; doi:10.1038/s43016-025-01130-1)

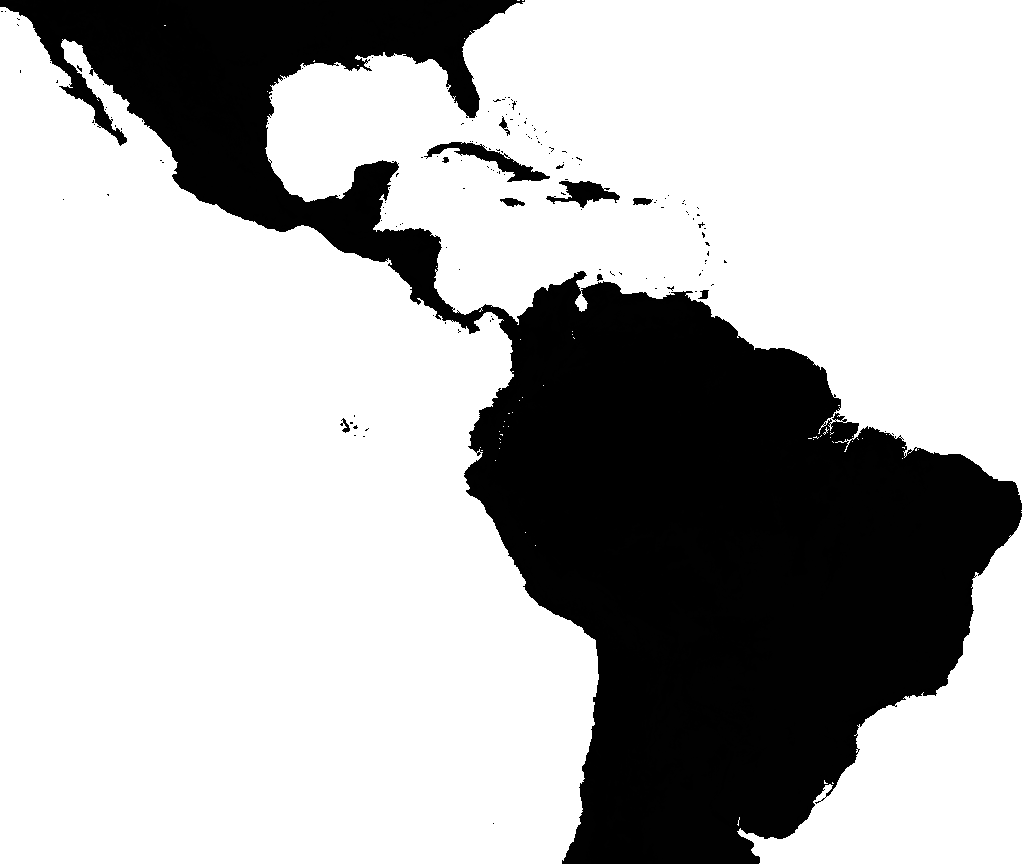

Supplement: Supplementary file 11 — GeoTIFF image files. [file 43016_2025_1130_MOESM11_ESM.zip › EDFig5_crop.tif]

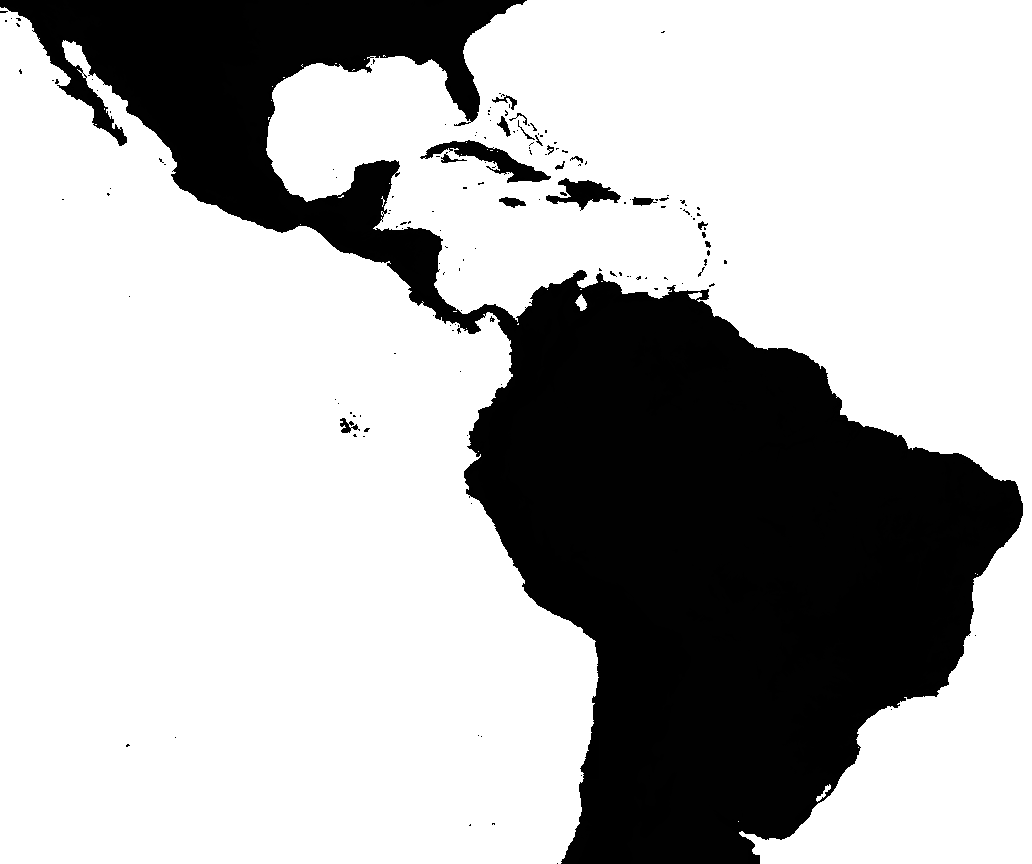

Supplement: Supplementary file 11 — GeoTIFF image files. [file 43016_2025_1130_MOESM11_ESM.zip › EDFig5_DEM.tif]

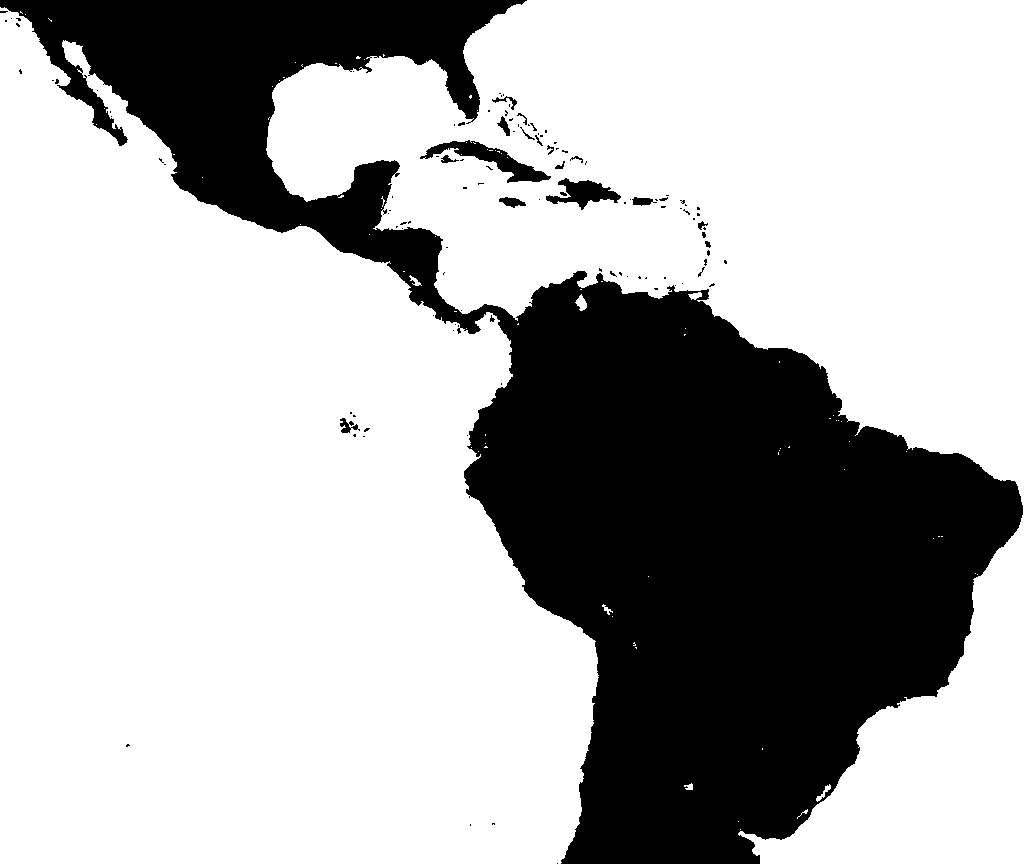

Supplement: Supplementary file 11 — GeoTIFF image files. [file 43016_2025_1130_MOESM11_ESM.zip › EDFig5_pH.tif]

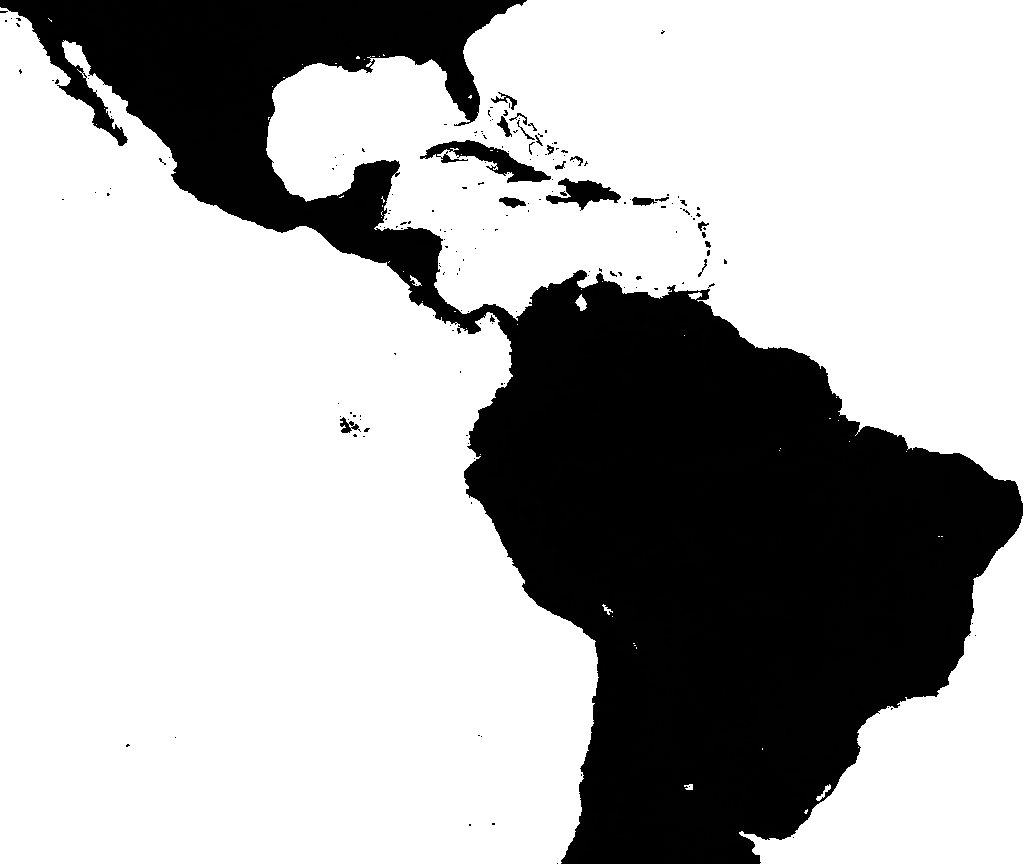

Supplement: Supplementary file 11 — GeoTIFF image files. [file 43016_2025_1130_MOESM11_ESM.zip › EDFig5_popdens.tif]

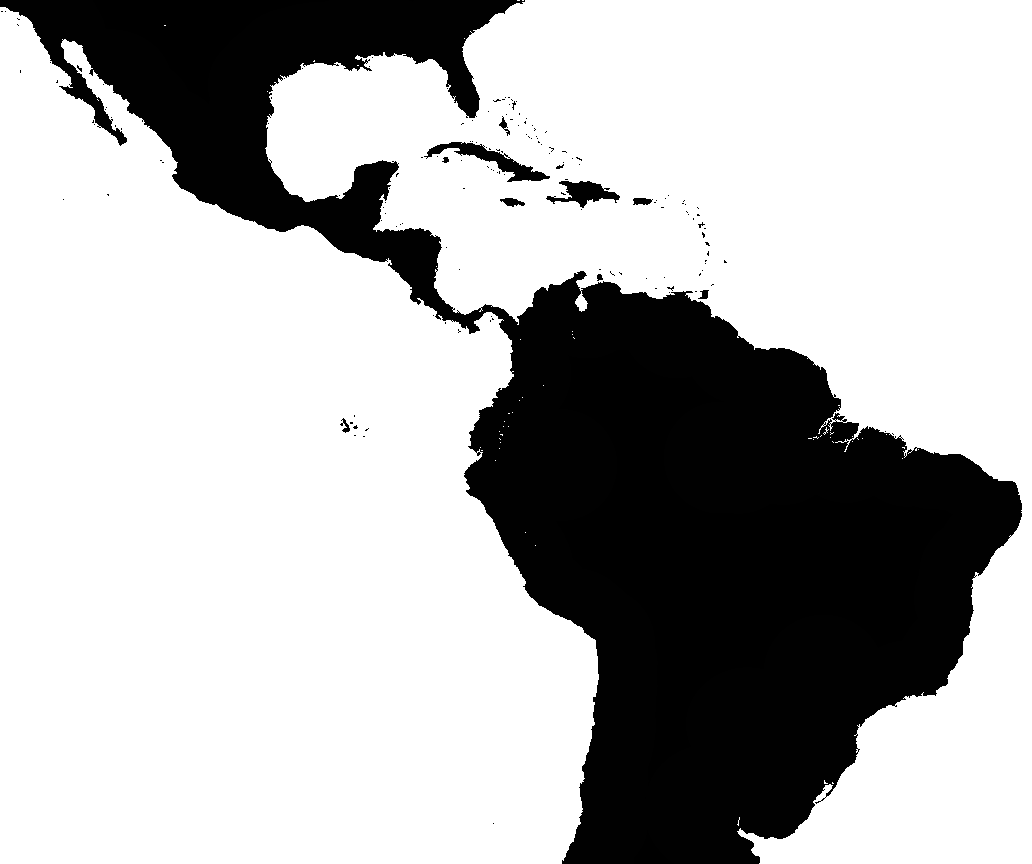

Supplement: Supplementary file 11 — GeoTIFF image files. [file 43016_2025_1130_MOESM11_ESM.zip › EDFig5_portdist.tif]

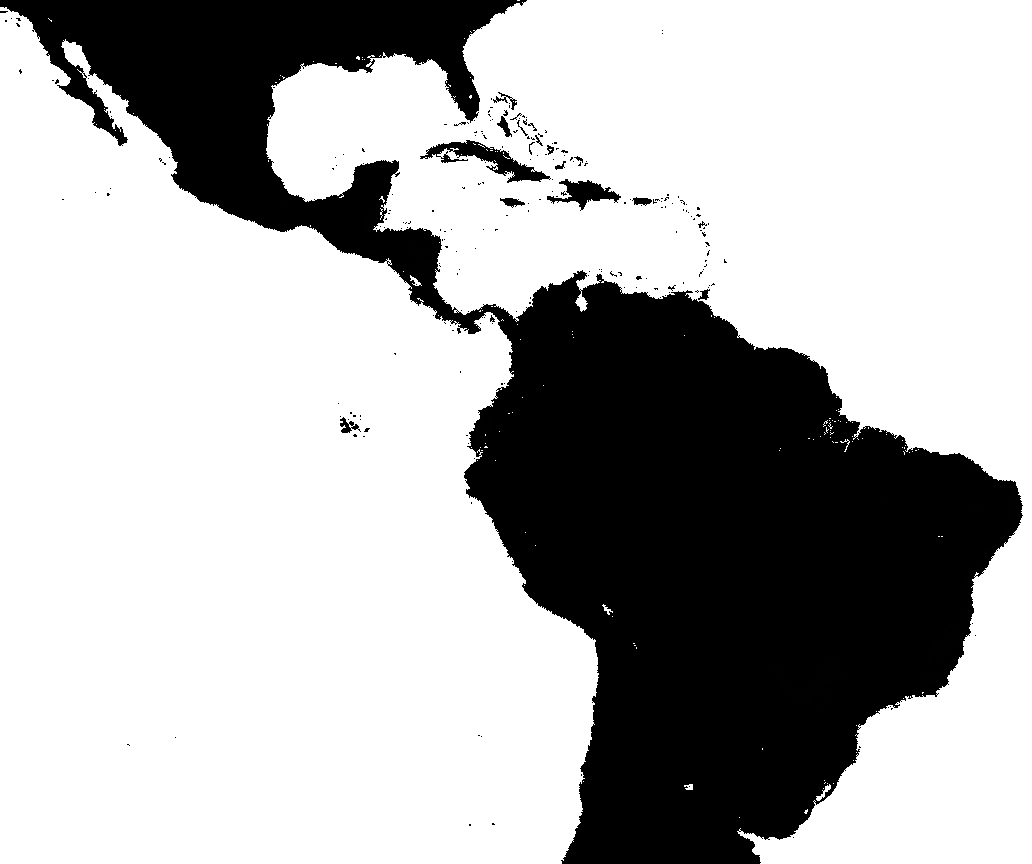

Supplement: Supplementary file 13 — GeoTIFF image files. [file 43016_2025_1130_MOESM13_ESM.zip › EDFig7_current.tif]

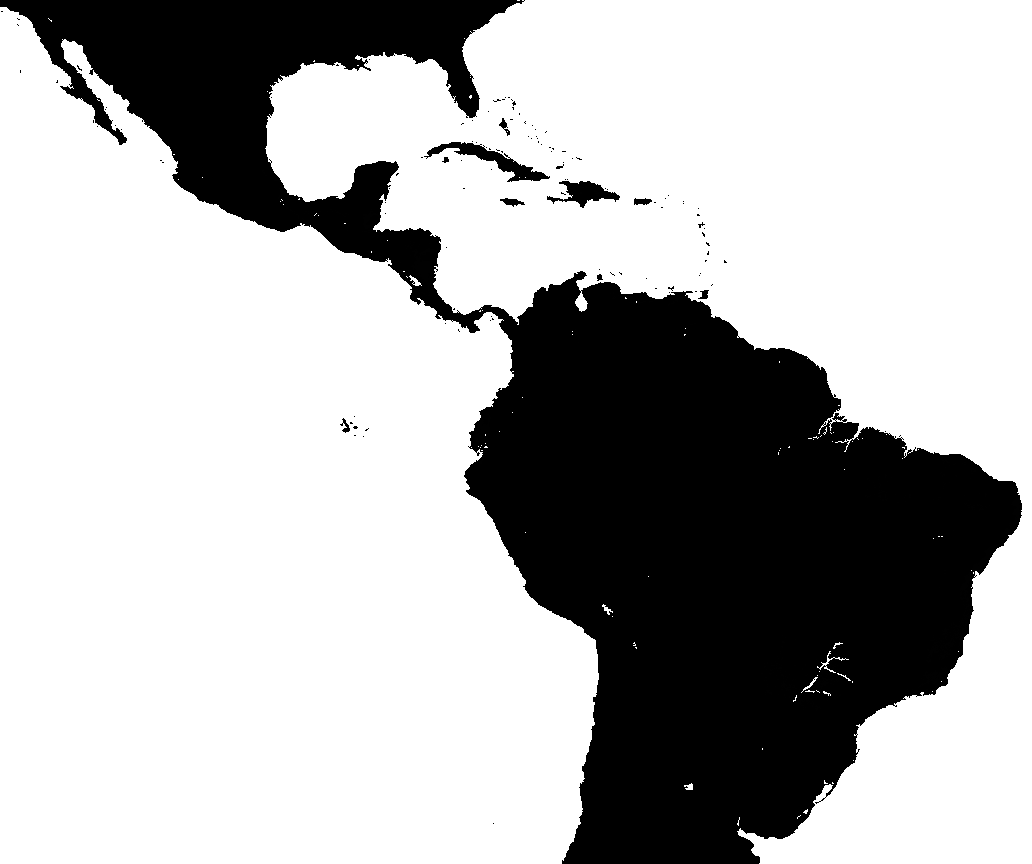

Supplement: Supplementary file 13 — GeoTIFF image files. [file 43016_2025_1130_MOESM13_ESM.zip › EDFig7_SSP1.tif]

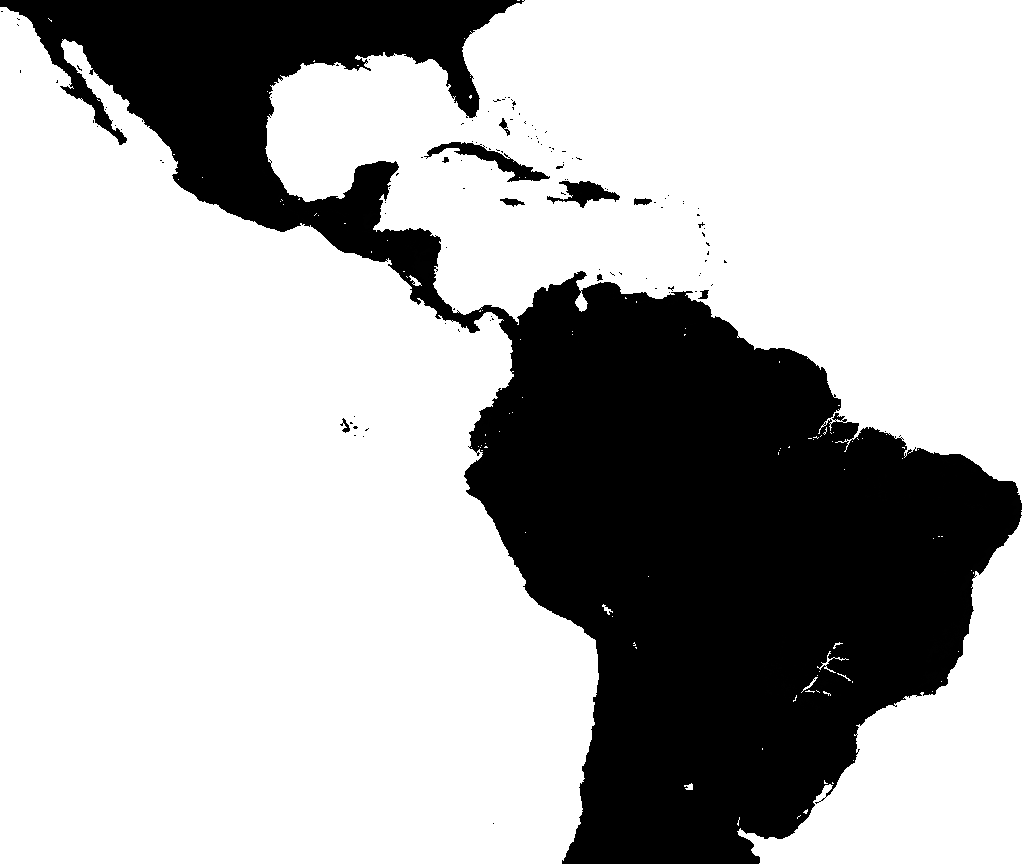

Supplement: Supplementary file 13 — GeoTIFF image files. [file 43016_2025_1130_MOESM13_ESM.zip › EDFig7_SSP2.tif]

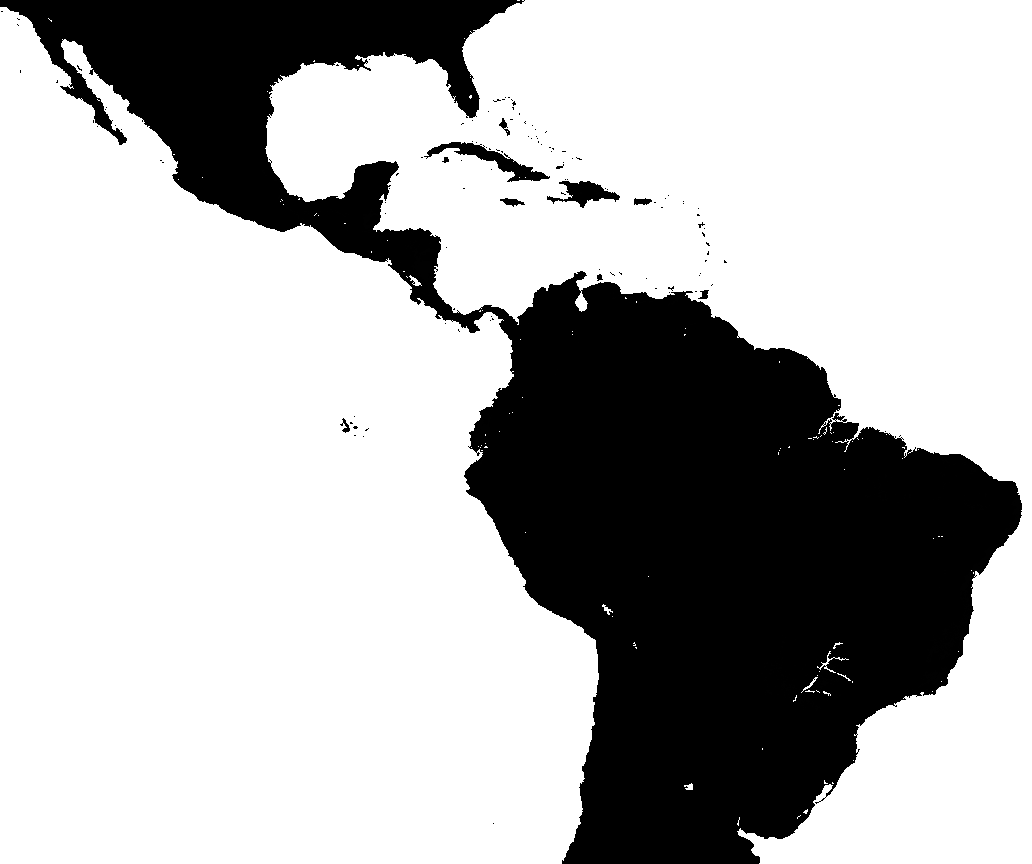

Supplement: Supplementary file 13 — GeoTIFF image files. [file 43016_2025_1130_MOESM13_ESM.zip › EDFig7_SSP3.tif]

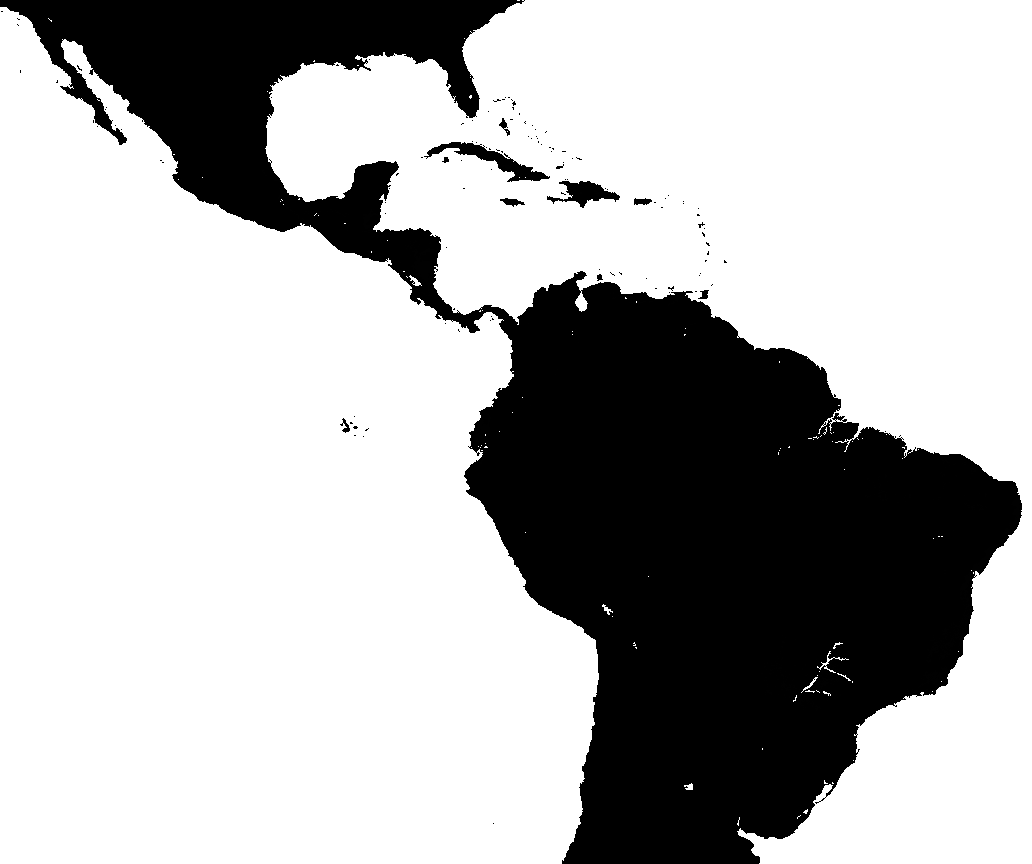

Supplement: Supplementary file 13 — GeoTIFF image files. [file 43016_2025_1130_MOESM13_ESM.zip › EDFig7_SSP4.tif]

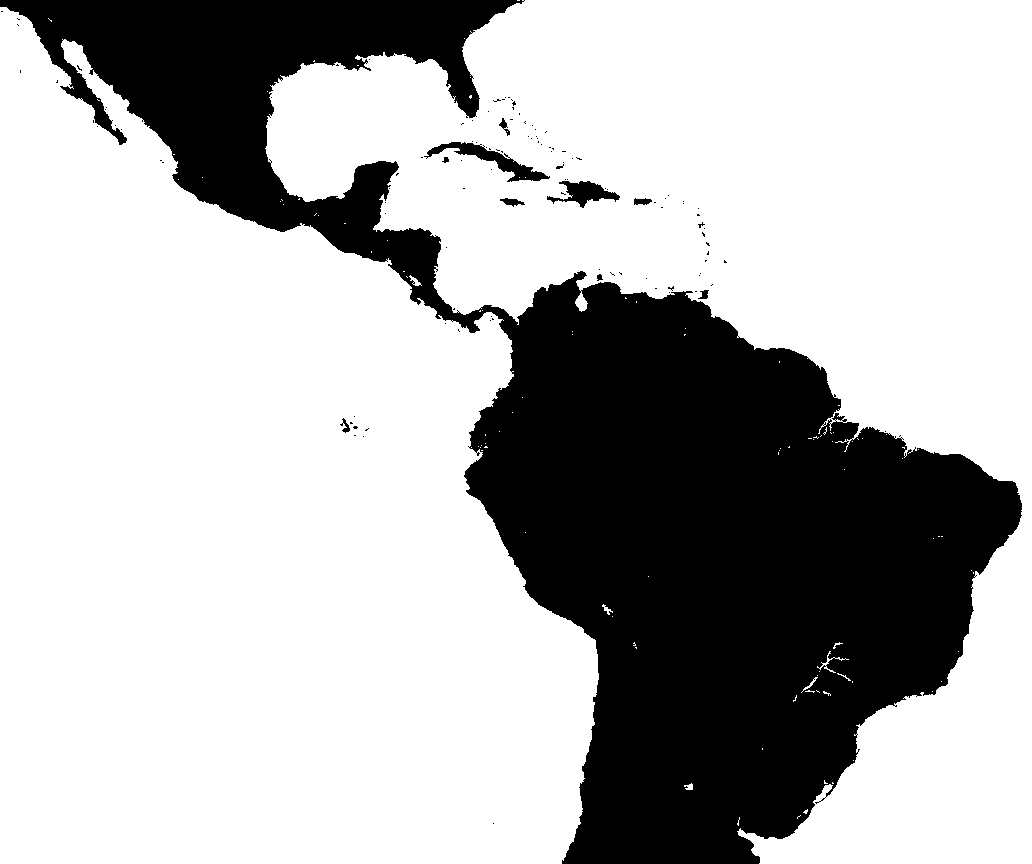

Supplement: Supplementary file 13 — GeoTIFF image files. [file 43016_2025_1130_MOESM13_ESM.zip › EDFig7_SSP5.tif]
